# The association between adolescent football participation and early adulthood depression

**DOI:** 10.1371/journal.pone.0229978

**Published:** 2020-03-10

**Authors:** Sameer K. Deshpande, Raiden B. Hasegawa, Jordan Weiss, Dylan S. Small

**Affiliations:** 1 Computer Science and Artificial Intelligence Laboratory, Massachusetts Institute of Technology, Cambridge, Massachusetts, United States of America; 2 Department of Statistics, University of Pennsylvania, Philadelphia, Pennsylvania, United States of America; 3 Population Studies Center, University of Pennsylvania, Philadelphia, Pennsylvania, United States of America; University of the Witwatersrand, SOUTH AFRICA

## Abstract

Concerned about potentially increased risk of neurodegenerative disease, several health professionals and policy makers have proposed limiting or banning youth participation in American-style tackle football. Given the large affected population (over 1 million boys play high school football annually), careful estimation of the long-term health effects of playing football is necessary for developing effective public health policy. Unfortunately, existing attempts to estimate these effects tend not to generalize to current participants because they either studied a much older cohort or, more seriously, failed to account for potential confounding. We leverage data from a nationally representative cohort of American men who were in grades 7–12 in the 1994–95 school year to estimate the effect of playing football in adolescent on depression in early adulthood. We control for several potential confounders related to subjects’ health, behavior, educational experience, family background, and family health history through matching and regression adjustment. We found no evidence of even a small harmful effect of football participation on scores on a version of the Center for Epidemiological Studies Depression scale (CES-D) nor did we find evidence of adverse associations with several secondary outcomes including anxiety disorder diagnosis or alcohol dependence in early adulthood. For men who were in grades 7–12 in the 1994–95 school year, participating or intending to participate in school football does not appear to be a major risk factor for early adulthood depression.

## Introduction

There has been growing concern about the long-term health consequences of playing American-style tackle football, driven in large part by high-profile suicides and case reports of chronic traumatic encephalopathy (CTE) among former players [1], increased risks or neurodegenerative disease [2], and associations between concussion history and cognitive impairment and depression later in life [3–5]. These concerns have led some medical professionals [6–8] and policy makers [9] to propose limiting or banning youth tackle football. Careful estimation of the short- and long-term consequences of playing youth and adolescent football can help physicians better advise families weighing the benefits and risks of football participation [10].

In the absence of a randomized trial, longitudinal studies are a compelling approach for studying these effects. We study the association of adolescent football participation and early adulthood depression using data from the National Longitudinal Study of Adolescent to Adult Health (Add Health) [11,12]. We conduct a matched observational study to estimate the effect of participation (or intention to participate) in middle and high school football on subjects’ scores on a variant of the Center for Epidemiological Studies Depression scale (CES-D) [13] measured in 2008. We also consider several concurrently measured secondary outcomes related to personality, substance abuse, and general health. We hypothesized that participation in football would be associated with higher CES-D scores (indicative of more depressive symptoms) and higher rates of diagnoses for depression, anxiety, and post-traumatic stress disorder, but not with differences in personality.

### Background and motivation

Strong associations between playing professional football and many adverse short- and long-term health outcomes have been reported in the literature. [1] reported that among a convenience sample containing 111 former professional players, 110 were diagnosed with CTE. Studying a set of 42 former professional players who were in their mid-50’s, [14,15] found that exposure to football related head trauma before age 12 was associated with cognitive impairment [14] and altered white matter structure [15]. While these studies are informative, they are potentially affected by strong selection bias through the use of volunteer participants. Among population-based studies of former professional players, [4] found that players with a history of concussions were 1.5 to 3 times more likely to be diagnosed with depression later in life than those without. Additionally, this cohort had elevated neurodegenerative mortality compared to the general US population [2], elevated all-cause, neurodegenerative, and cardiovascular mortality compared to professional baseball players [16], but similar mortality to replacement players who were temporarily hired to play during a league-wide strike [17].

Since the vast majority of adolescent participants do not play collegiately or professionally, it is unknown whether they suffer the same risks as professional players. For instance, although a single season of youth tackle football can result in detectable acute white matter changes in the brain [18–20], the long-term implications of these changes are yet to be established. Furthermore, there are positive health and psychological benefits of youth sports participation including reduced social anxiety [21], higher self-esteem [22], improved psychological resilience [23], and greater life satisfaction [24]; see [25,26] and references therein for a comprehensive review on the psychological and social health benefits of youth sports participation. This motivates us to study the following questions: to what extent, if any, do these benefits of sports participation extend specifically to football players? And do the potential harms associated with repetitive head trauma associated with football participation outweigh these potential benefits?

In the absence of randomized trials, longitudinal studies arguably offer the most promise for answering these questions. However, the evidence from existing longitudinal studies is mixed and methods vary considerably. Using data from the on-going Longitudinal Examination to Gather Evidence of Neurodegenerative Disease (LEGEND) [27,28], [29] observed a dose-response relationship between cumulative head impacts and risk for later-life cognitive impairment and depression. Studying the same sample, [30] reported that exposure to football before age 12 was associated with increased odds of cognitive and neuropsychiatric impairment. A more recent study [31] of the same sample, however, found that age of first exposure was not associated with CTE pathology. These studies are limited by the use of volunteer participants and their retrospective design. In contrast, [32–34] considered population-level random samples and reported that high school football participation was not associated with elevated rates of neurodegenerative disease [32,33] and cognitive decline and depression [34]. Unfortunately, these latter three studies all considered cohorts of men who attended high school in the 1940’s and 1950’s. Further, [29,32,33] did not control for any confounders, limiting the ability to draw causal conclusions.

In this study, we aim to overcome methodological limitations of these longitudinal studies that prevent the generalization of their findings. In particular, we use data from a recent longitudinal study (Add Health) that has prospectively followed a randomly selected nationally representative sample since adolescence. Parallel to but independently of our analysis, [35] analyzed data from Add Health and found that participating in school football was not associated with impaired cognitive ability, increased depressive symptoms, or increased suicidal ideation. While [35] controlled for only six potential confounders, we are able to control for over a hundred potential confounders that were measured in adolescence through a careful matching procedure. This ensures that we only compare outcomes among the most comparable subjects in our study.

## Methods

This matched observational study analysis restricted use data from the Add Health study. The data was fully anonymized by Add Health prior to our access and analysis. Additional details on the data and its availability are available in S1 Appendix. The University of Pennsylvania’s Institutional Review Board approved the research protocol. The matches were constructed prior to looking at the outcome data and were posted along with our protocol online to arXiv (identifier: arXiv:1808.03934), as recommended by [36].

### Study population

Add Health enrolled a nationally representative sample of 12,105 American adolescents who were in grades 7–12 during the 1994–95 academic year and conducted follow-ups in 1996, 2001–02 and 2008. We consider athletic participation in 1994–95, when respondents were asked whether they are participating this year or plan to participate later in the school year in various sports. Either participating in or planning to participate in a sport will henceforth be called participation; similar measures of athletic participation in Add Health have been used previously [37–39]. We consider outcomes in 2008, when subjects were aged 24–32. Add Health contains a rich set of baseline variables measured in adolescence allowing for careful adjustment for potential confounders. Details about the Add Health design have been published previously [11,12].

**Fig 1:**
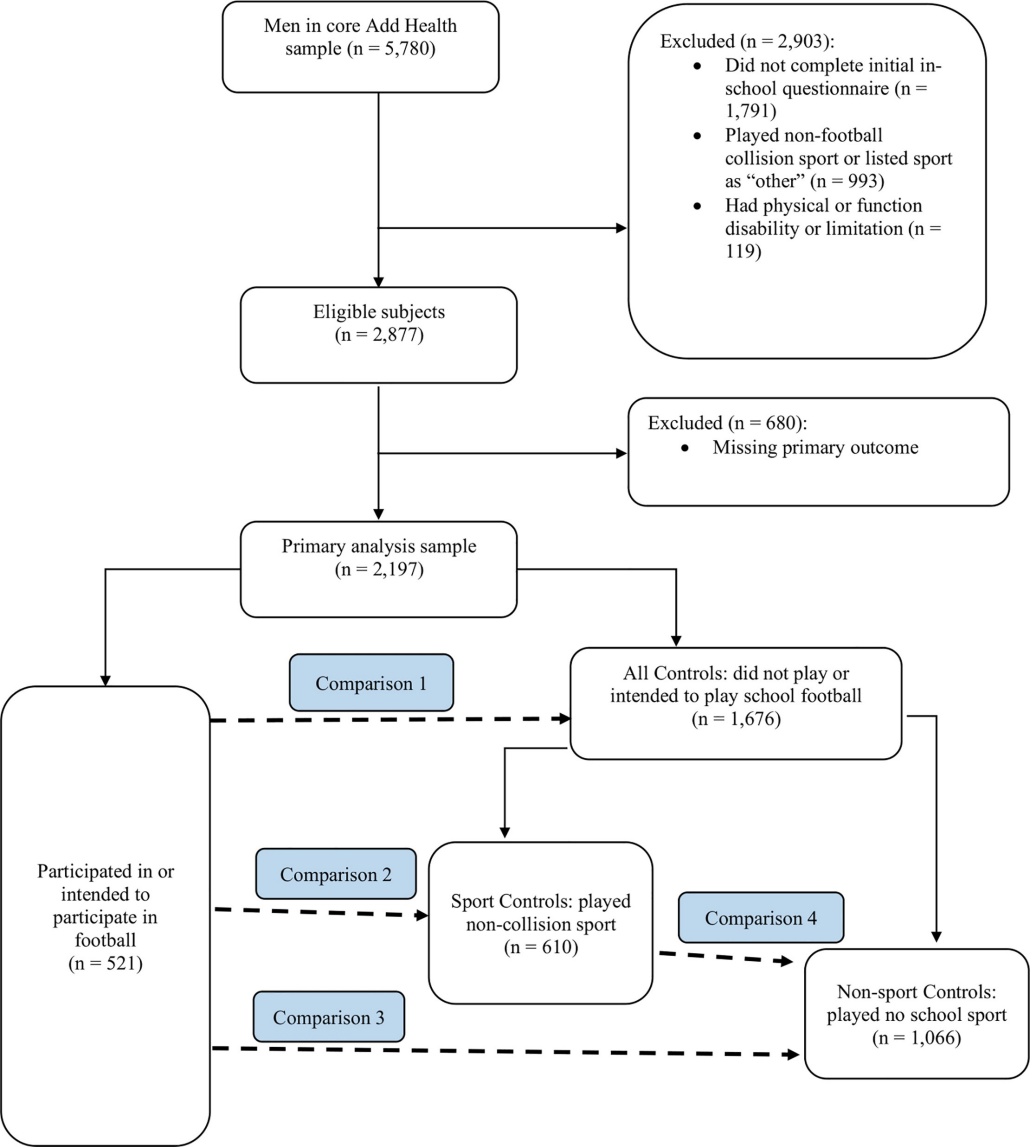
Flow diagram outlining inclusion and exclusion criteria, division of control group into two subsets, and schematic of four comparisons.

Athletic participation information was unavailable for 1,791 (31.0%) of the 5,780 men in the Add Health sample. Of the remaining 3,989, we excluded 993 (33.2%) who indicated they participated in sports with a high incidence of head trauma (soccer, hockey, and wrestling) in that academic year and a further 119 (3.0%) who had a physical or functional disability. 680 (23.5%) of the remaining 2,887 men eligible for our study were missing primary and secondary outcomes and were excluded. Fig 1 summarizes these exclusions. Further details about the eligibility and inclusion criteria are in the supplemental S1 Appendix and Tables A—B in S1 Appendix. Of the final 2,197 subjects, 521 (23.7%) participated in football.

### Primary and secondary outcomes

Our primary outcome is the score on a five-item variant of the full CES-D [13] scale recommended by [40], scores ranging from 0 (least depressed) to 15 (most depressed). Secondary outcomes include binary indicators of alcohol, nicotine, and cannabis dependence or abuse, and indicators of depression, anxiety, and post-traumatic disorder diagnoses in adulthood. Previous research suggests that personality may contribute substantially to subjective well-being and mental health [41–44]. In particular, neuroticism, extraversion, and conscientiousness have all been associated with depression [41,42]. Motivated by this, we include among our secondary outcomes scores from an inventory of the “Big 5” personality dimensions [45] (agreeableness, conscientiousness, extraversion, neuroticism, and openness), which were measured in 2008 using a validated mini International Personality Item Pool (mini-IPIP) [46].

### Statistical analysis

#### Attrition analysis

Nearly 25% of eligible subjects were excluded because they were missing the primary and most secondary outcomes. To examine whether playing football increased the likelihood of attrition from Add Health, we fit a logistic regression to predict availability of the primary outcome using the exposure indicator and several baseline variables related to family background and adolescent health, personality, and patterns of alcohol, cigarette, and drug use. A full list of these variables is available in S1 Appendix.

#### Matching methodology

To control for potential confounding variables, we used variable-ratio matching [47,48] to form sets containing one football player and one or more controls that balance the distribution of baseline variables (the same as in our attrition analysis) between football players and controls. To achieve a good compromise between overall covariate balance and similarity of matched subjects, we matched using a propensity score-calipered rank-based Mahalanobis distance between the baseline covariates of each pair of exposed and control subject [49].

We also considered two subgroups as alternative control groups: those controls who played a sport with low incidence of head trauma like basketball and tennis (sport controls) and those controls who did not play any school sport (non-sport controls). These two subgroups may differ along unmeasured dimensions like personality or fitness that may affect our outcomes. Comparability of the two subgroups of controls would mitigate concern about these unmeasured confounders [50,51]. A convincing study of an effect of playing football specifically (not just playing sports generally) would show consistent evidence across comparisons of football players with all controls, sport controls, and non-sport controls. In all, we perform four comparisons–football vs all controls, football vs sport-controls, football vs non-sport controls, and sport controls vs non-sport controls. We construct a separate match for each comparison.

Our objective in matching is to achieve standardized differences between the two matched groups on baseline covariates below 0.2 standard deviations, as biases due to residual imbalance this small may be removed by regression adjustment [52–54]. Matching was performed prior to analysis of the outcome data between April 1 and August 10, 2018.

#### Outcome analysis

Though matching can help eliminate some bias from comparing outcomes of football players to those of controls, some bias remains due to residual covariate imbalances. To further reduce this bias, matching can be combined with regression adjustment, comparing the residuals of the exposed subjects and their matched controls [52–54]. For regression adjustment, we use Bayesian Additive Regression Trees (BART), a nonparametric technique that has shown acuity in automatically detecting non-linearities and interactions [55]. We assessed effect sizes as follows: between 0.01 and 0.2 SDs for very small effects, between 0.2 and 0.5 SDs for small effects, between 0.5 and 0.8 SDs for medium effects, between 0.8 and 1.2 SDs for large effects, and over 1.2 SDs for very large effects [56,57]. For the CES-D score, these cut-offs (on the absolute scale) were 0.02 for very small effects, 0.46 for small effects, 1.14 for medium effects, 1.82 for large effects, and 2.74 for very large effects. For the binary secondary outcomes, we fit conditional logistic regression models and reported outcomes on the odds ratio (OR) scale. The cut-off for small effect sizes was 1.5 on the OR scale [58].

#### Ordered hypothesis testing

To perform the aforementioned comparisons with different control groups without losing power due to multiple testing, we used the same ordered testing procedure of [34]. For the sake of completeness, we also report results from each comparison even if it is not reached in the ordered testing procedure. In such cases, the confidence intervals are left unadjusted for multiple testing and are designated as such.

## Results

### Attrition analysis

After adjusting for baseline variables, football players’ missingness of CES-D score in 2008 was not statistically significantly different from controls (OR = 0.94, 95% CI: 0.73, 1.19). Further the 95% confidence interval only contains very small effect sizes, somewhat mitigating concern that our analysis is substantially affected by differential attrition.

### Matching

Table 1 shows a subset of standardized differences from matching football players with all controls. Prior to matching, compared to all controls, football players were about 4.6 kg heavier, more likely to identify as black or African-American and rate their health as “excellent”, and less likely to never experience joint or muscle pain or to smoke regularly. The standardized differences along these variables were all greater than 0.2 SDs in absolute value, revealing unacceptable balance prior to matching. After matching, these standardized differences were all less than 0.2 SDs, indicating that the matched football players were much more comparable to the matched controls. Importantly for our mental health outcomes, we find that matched football players and matched controls reported in adolescence similar frequencies of headaches, dizziness, and trouble sleeping, and similar patterns of alcohol and cigarette consumption. The other three matches were similar (Tables D–G in S1 Appendix).

**Table 1:** Comparison of average baseline variables related to demographics, educational aspirations, substance use, general health, and self-perception for football players vs all controls, before and after matching.

	Before Matching		After Matching		Standardized Difference	
Variable	Football	All Controls^a^	Football	All Controls^a^	Before	After
Age in 2008 (yrs)	28.76	29.06	28.71	28.71	-0.173	0
Weight in 1994–95 (kg)	72.61	68.04	71.53	70.44	0.263	0.063
Height in 1994–95 (cm)	173.96	173.93	173.63	173.38	0.002	0.024
Self-reported race^b^						
White (%)	66.03 (344/521)	72.55 (1216/1676)	67.79 (303/447)	66.34 (742/1034)	-0.175	0.039
Black (%)	27.45 (143/521)	17.72 (297/1676)	25.5 (114/447)	26.75 (212/1034)	0.282	-0.036
Native American (%)	2.69 (14/521)	2.63 (44/1676)	2.46 (11/447)	2.32 (24/1034)	0.005	0.011
Asian (%)	2.69 (14/521)	4.83 (81/1676)	2.46 (11/447)	2.81 (42/1034)	-0.155	-0.025
Other (%)	4.99 (26/521)	6.26 (105/1676)	4.92 (22/447)	5.34 (49/1034)	-0.072	-0.024
Desire to go to college						
Low (%)	3.26 (17/521)	5.43 (91/1676)	2.68 (12/447)	2.5 (33/1034)	-0.144	0.012
Medium (%)	7.49 (39/521)	12.17 (204/1676)	7.38 (33/447)	8.09 (108/1034)	-0.212	-0.032
High (%)	73.7 (384/521)	62.35 (1045/1676)	73.6 (329/447)	72.59 (703/1034)	0.317	0.028
Likelihood to go to college (%)						
Low (%)	3.84 (20/521)	6.38 (107/1676)	3.13 (14/447)	2.98 (37/1034)	-0.157	0.009
Medium (%)	14.2 (74/521)	16.17 (271/1676)	13.2 (59/447)	13.29 (138/1034)	-0.07	-0.003
High (%)	53.74 (280/521)	47.2 (791/1676)	55.03 (246/447)	54.74 (535/1034)	0.165	0.007
Ever tried cigarette smoking (%)	53.55 (279/521)	56.09 (940/1676)	51.23 (229/447)	49.65 (508/1034)	-0.064	0.04
Smoked regularly (%)	14.01 (73/521)	20.47 (343/1676)	12.08 (54/447)	12 (135/1034)	-0.226	0.003
Ever drank alcohol (%)	55.09 (287/521)	55.01 (922/1676)	53.02 (237/447)	49.72 (484/1034)	0.002	0.083
How often subject drank alcohol in past year						
3–5 days/week (%)	2.88 (15/521)	3.28 (55/1676)	2.24 (10/447)	2.48 (20/1034)	-0.03	-0.018
Once a month or less (%)	10.56 (55/521)	11.1 (186/1676)	10.07 (45/447)	9.17 (103/1034)	-0.022	0.036
Not applicable^c^ (%)	45.11 (235/521)	45.35 (760/1676)	46.98 (210/447)	50.28 (550/1034)	-0.005	-0.066
Self-reported rating of general health						
Excellent (%)	39.35 (205/521)	30.91 (518/1676)	38.7 (173/447)	37.59 (340/1034)	0.22	0.029
Very good (%)	39.54 (206/521)	39.56 (663/1676)	39.82 (178/447)	40.79 (425/1034)	0	-0.025
Good (%)	16.89 (88/521)	24.58 (412/1676)	17 (76/447)	17.57 (227/1034)	-0.25	-0.019
Fair (%)	3.84 (20/521)	4.42 (74/1676)	4.03 (18/447)	3.74 (39/1034)	-0.037	0.018
Poor(%)	0.38 (2/521)	0.48 (8/1676)	0.45 (2/447)	0.31 (3/1034)	-0.019	0.028
How often in past week subject exercised						
Not at all (%)	15.55 (81/521)	23.21 (389/1676)	14.99 (67/447)	15.82 (217/1034)	-0.257	-0.028
5+ times (%)	32.25 (168/521)	23.63 (396/1676)	30.43 (136/447)	28.52 (247/1034)	0.236	0.052
How often in past year has subject had						
Headaches						
A few times (%)	70.44 (367/521)	67.18 (1126/1676)	70.25 (314/447)	66.23 (702/1034)	0.089	0.11
Almost every day (%)	2.5 (13/521)	2.98 (50/1676)	2.24 (10/447)	1.64 (26/1034)	-0.039	0.047
Dizziness						
Never (%)	69.1 (360/521)	65.21 (1093/1676)	68.9 (308/447)	68.93 (704/1034)	0.105	-0.001
Once a week (%)	4.22 (22/521)	3.64 (61/1676)	3.58 (16/447)	2.09 (25/1034)	0.037	0.094
Muscle or joint pain						
Never (%)	11.13 (58/521)	20.17 (338/1676)	10.96 (49/447)	13.57 (181/1034)	-0.34	-0.098
Once a week (%)	28.6 (149/521)	20.41 (342/1676)	28.86 (129/447)	24.26 (207/1034)	0.233	0.131
Trouble Sleeping						
Never (%)	44.15 (230/521)	42.96 (720/1676)	44.3 (198/447)	44.13 (449/1034)	0.03	0.004
Almost every day (%)	4.61 (24/521)	7.04 (118/1676)	3.8 (17/447)	5.63 (57/1034)	-0.139	-0.104
Seriously contemplated suicide (%)	9.4 (49/521)	10.2 (171/1676)	7.38 (33/447)	6.49 (78/1034)	-0.034	0.038
How much subject agrees that						
You are physically fit						
Strongly agree (%)	42.61 (222/521)	31.38 (526/1676)	40.94 (183/447)	43.1 (353/1034)	0.289	-0.056
Disagree (%)	1.73 (9/521)	6.03 (101/1676)	2.01 (9/447)	2.51 (51/1034)	-0.34	-0.039
Strongly disagree (%)	0 (0/521)	0.72 (12/1676)	0 (0/447)	0.07 (3/1034)	-0.233	-0.024
You feel socially accepted						
Strongly agree (%)	38.2 (199/521)	31.8 (533/1676)	36.24 (162/447)	38.89 (355/1034)	0.167	-0.069
Disagree (%)	1.15 (6/521)	3.76 (63/1676)	0.89 (4/447)	1.76 (28/1034)	-0.255	-0.084

^a^ Before matching, control values are unweighted. After matching, control values are weighted according to the composition of the matched sets (Table C in S1 Appendix).

^b^ Subjects were allowed to report more than one race.

^c^ These subjects were not asked this question as they had previously indicated they had not consumed alcohol

### Primary analysis

Table 2 reports the estimated effect of playing football on CES-D scores in 2008. After adjusting for covariates, football players’ CES-D scores were not significantly different from matched controls’ scores (CI: -0.52, 0.02). For this comparison, the cut-off for a small effect is 0.46 and negative values correspond to reporting fewer depressive symptoms. Though the 95% CI contains small beneficial effect sizes (i.e. negative differences in CES-D score), it excludes small harmful effect sizes.

**Table 2:** Effect of playing football relative to each control condition.

Comparison	Effect (95% CI)	Small/Large Effect Cut-off	P-value
Comparison 1 (Football vs all controls)	-0.26 (-0.52, 0.02)	0.46 / 1.82	0.06
Comparison 2 (Football vs sport controls)	-0.24 (-0.57, 0.02) ^a^	0.43 / 1.72	0.11 ^a^
Comparison 3 (Football vs non-sport controls)	-0.21 (-0.47, 0.11) ^a^	0.45 / 1.80	0.22 ^a^
Comparison 4 (Sport controls vs non-sport controls)	0.02 (-0.25, 0.27) ^a^	0.44 / 1.76	0.88 ^a^

^a^ Confidence interval and p-value are unadjusted for multiple comparisons.

Similarly, football players’ CES-D scores were not significantly different than matched sport controls and non-sport controls’ scores (football vs sport controls: unadjusted 95% CI: -0.57, 0.02; football vs non-sport controls: unadjusted 95% CI: -0.47, 0.11). Finally, sport controls’ CES-D scores were not significantly difference than those of the non-sport controls (unadjusted 95% CI: -0.25, 0.27).

### Secondary analysis

Table 3 reports the estimated effect of playing football on secondary outcomes when comparing football players to all controls. Table H–J in S1 Appendix are analogs for the remaining comparisons. Effects on binary outcomes are reported on the odds ratio (OR) scale while results for continuous outcomes are reported on the absolute scale.

**Table 3:** Effects of playing football on secondary outcomes compared to all controls.

Outcome	Effect (95% CI)	Small/Large Effect Cut-off	Unadjusted *P* Value (Adjusted *P* Value ^a^)
General Health Outcomes			
Daily Smoker (y/n)	0.83 (0.61, 1.13)	1.50 / 5.00	0.24 (0.77)
Physically Active in 2008 (y/n)	0.8 (0.55, 1.16)	1.50 / 5.00	0.24 (0.77)
Diagnosis of following after age 18			
High cholesterol or triglycerides (y/n)	1.20 (0.77, 1.89)	1.50 / 5.00	0.42 (0.81)
High Blood Pressure or Hypertension (y/n)	0.85 (0.57, 1.27)	1.50 / 5.00	0.42 (0.81)
High blood sugar or diabetes (y/n)	0.46 (0.17, 1.28)	1.50 / 5.00	0.14 (0.77)
Heart disease (y/n)	0.52 (0.06, 4.74)	1.50 / 5.00	0.56 (0.90)
Migraine Headaches (y/n)	0.94 (0.45, 1.97)	1.50 / 5.00	0.87 (0.90)
Depression (y/n)	0.76 (0.43, 1.34)	1.50 / 5.00	0.34 (0.77)
PTSD (y/n)	1.17 (0.46, 2.97)	1.50 / 5.00	0.74 (0.90)
Anxiety or Panic Disorder (y/n)	0.93 (0.54, 1.62)	1.50 / 5.00	0.80 (0.90)
Seriously contemplated suicide in past year (y/n)	0.78 (0.40, 1.52)	1.50 / 5.00	0.47 (0.83)
Substance Dependence/Abuse Outcomes			
Nicotine (y/n)	0.75 (0.50, 1.13)	1.50 / 5.00	0.17 (0.77)
Alcohol (y/n)	1.15 (0.89, 1.49)	1.50 / 5.00	0.29 (0.77)
Cannabis (y/n)	0.83 (0.58, 1.17)	1.50 / 5.00	0.28 (0.77)
Personality Scale Scores			
Cohen Perceived Stress	0.05 (-0.32, 0.4)	0.89 / 3.54	0.90 (0.90)
Anxiety Scale	-0.05 (-0.36, 0.3)	0.73 / 2.93	0.86 (0.90)
Anger/Hostility Scale	0.06 (-0.26, 0.38)	0.56 / 2.26	0.71 (0.90)
Optimism Scale	0.04 (-0.22, 0.32)	0.64 / 1.61	0.75 (0.90)
“Big 5” Personality Traits			
Agreeableness	-0.13 (-0.43, 0.14)	0.65 / 2.62	0.33 (0.77)
Conscientiousness	0.19 (-0.12, 0.51)	0.80 / 1.99	0.30 (0.77)
Extraversion	0.04 (-0.32, 0.38)	0.76 / 3.02	0.84 (0.90)
Neuroticism	-0.04 (-0.37, 0.22)	0.68 / 2.73	0.77 (0.90)
Openness	-0.5 (-0.9, -0.16)	1.44 / 5.78	0.00 (0.02)

^a^ *P* values were adjusted using the Benjamini-Hochberg procedure

Compared to all controls, football players in our study were not significantly more or less likely than matched controls to be daily smokers (OR = 0.83; unadjusted 95% CI: 0.61, 1.13), to have been diagnosed with dependence to or abuse of nicotine (OR = 0.75; unadjusted 95% CI: 0.50, 1.13), cannabis (OR = 0.83; unadjusted 95% CI: 0.58, 1.17), or alcohol (OR = 1.15; unadjusted 95% CI: 0.89, 1.49). Our findings are similar when comparing football players to each sub-set of controls (Tables H–J in S1 Appendix).

In line with our primary analysis, football players were not significantly more or less likely than all controls to have been diagnosed with depression (OR = 0.76; unadjusted 95% CI: 0.43, 1.34), migraine headaches (OR = 0.94; unadjusted 95% CI: 0.45, 1.97), anxiety (OR = 0.93; unadjusted 95% CI: 0.54, 1.62), or PTSD (OR = 1.17; unadjusted 95% CI: 0.46, 2.97). Furthermore, football players were not significantly more or less likely to have seriously considered suicide (OR = 0.78; unadjusted 95% CI: 0.4, 1.52).

The unadjusted confidence intervals for the effect of playing football on the agreeableness, conscientiousness, extraversion, and neuroticism scales each contained both positive and negative effects. However, none of these intervals contained even small effect sizes (Table 3). For the openness scale, football players scored 0.5 points lower on average and the corresponding interval contained only negative effects (unadjusted 95% CI: -0.9, -0.16). We note that openness is largely unrelated to anxiety, depression, and substance abuse disorders [41] and that this interval also does not contain even small effect sizes.

## Discussion

Our study suggests potential adverse effects of youth football participation might not manifest in early adulthood. Specifically, we did not find evidence that participation in middle or high school football had a harmful effect on depression in early adulthood among a nationally representative sample of American men who were in grades 7–12 in the 1994–95 school year. Moreover, we did not find evidence to suggest that participation in middle or high school football increased the likelihood of alcohol, cannabis, or nicotine dependence or abuse in early adulthood, on average. Finally, we did not find evidence that playing football had even a small effect on the “Big 5” personality dimensions; in fact, football players and controls were quite similar along the dimensions most associated with depression (conscientiousness, extraversion, and neuroticism).

Our primary finding is broadly consistent with [34], who reported a small, statistically significant beneficial effect of playing football on CES-D scores at age 65, [59], who found school-sport participation was associated with lower depressive symptoms, perceived stress, and higher self-rated mental health, and [35], who reached similar conclusions as us in a concurrent but methodologically distinct analysis of the same Add Health cohort. Our findings also accord with the broader literature on the benefits of adolescent physical activity [60]: regular physical activity during adolescence may decrease the risk of diabetes [61] and obesity [62], improve psychological and social health [26], and may even protect against later-life neurodegeneration [63]. Additionally, our finding that any adverse adolescent football participation might not manifest in early adulthood is similar to [64] finding that participating in tackle football before age 12 may not result in short-term neurocognitive deficits in college.

### Strengths and limitations

Our study overcomes many of the design and methodological limitations that prevent generalizing the findings of existing longitudinal studies about the effects of playing football. While we were able to control for many important potential confounders, it is possible that there are unmeasured confounders. The similarity of our results across comparisons with multiple control groups mitigates this concern somewhat.

The fact that nearly 25% of eligible participants lacked primary outcomes raises some concern about potential attrition bias. However, we found that football players were not significantly more or less likely to be missing the primary outcomes, somewhat mitigating this concern. Since our outcomes were constructed from survey responses, our results might be affected by response bias.

Perhaps of more concern, the Add Health dataset only recorded whether subjects were currently participating in or intended to participate in various school sports. While these measures has been used as a proxy for sports participation before [37–39], it is possible that some subjects in our football group did not end up playing and vice versa.

It is almost certain that some subgroup of football players in our study experienced higher levels of head trauma or suffered multiple concussions and therefore might be at an elevated risk of depression and other neurological dysfunction. In fact, several studies have found that history of multiple concussions may have long-term cognitive and behavioral consequences [3,4,29,65] and that the frequency and severity of head impacts vary by position played [66]. Unfortunately, the Add Health dataset did not contain detailed information about subjects’ injury history or football position played, limiting our ability to study potential subgroup effects in this paper. Identifying potential subgroups and estimating the effect within these subgroups from observational studies is an important direction for future research.

Though we did not find that playing football was harmful on average, it is not without considerable risks. Our results should not impede the development and adoption of common-sense measures like improved concussion management protocols, eliminating kick-offs [67], or age-restrictions on tackling [68].

## Supporting information

S1 Appendix Appendix contains additional information about the eligibility and inclusion criteria, statistical analyses, availability of the data and analysis code, a full list of all baseline variables used in our matching, and additional tables. (DOCX)
