# Supplementary material for: The association between adolescent football participation and early adulthood depression
Source: PLoS One. 2020 Mar 10;15(3):e0229978. doi: 10.1371/journal.pone.0229978 (PMC7064245; doi:10.1371/journal.pone.0229978)
Supplement: S1 Appendix — (DOCX) [file pone.0229978.s001.docx]

**S1 Appendix**

This appendix contains additional information about the eligibility and inclusion criteria, statistical analyses, availability of the data and analysis code, and a full list of all baseline variables used in our matching. It also contains several additional tables, listed below.

**Table A. Sports participation information.**

**Table B. Summary of responses used to determine whether Add Health subject had a physical limitation or disability.**

**Table C. Composition of matched sets.**

**Table D. Comparison of average baseline variables for football players vs all controls.**

**Table E. Comparison of average baseline variables for football Players vs sport controls.**

**Table F. Comparison of average baseline variables for football players vs non-sport controls.**

**Table G. Comparison of average baseline variables for sport controls and non-sport controls.**

**Table H. Effects of playing football on secondary outcomes compared to sport controls.**

**Table I. Effects of playing football on secondary outcomes compared to non-sport controls**

**Table J. Effects of playing a non-collision sport vs not playing any sport on secondary outcomes.**

**Eligibility and Inclusion Criterion**

The National Longitudinal Study of Adolescent to Adult Health (Add Health) is a nationally representative and longitudinal study of American adolescents. Study participants were drawn from a probability sample of 80 high schools and 52 middle schools which were representative of the US (grades 7-12) in 1994-1995 [1]. More than 90,000 participants were invited to complete an in-school survey during the baseline years 1994-1995, from which a random sample of over 20,000 students were selected to complete an in-home survey in 1995 (Wave I). Participants were followed-up in 1996 (Wave II), 2001 -- 02 (Wave III), and 2008 (Wave IV) to provide data on sociodemographic characteristics, household characteristics, school participation, and extracurricular activities, including participation in contact sports. For more detailed information on the Add Health study and its design, please see [1,2].

In all, there are 5,780 men in the core Add Health sample, of whom 3,989 had completed the in-school questionnaire. This questionnaire collected measurements on a range of topics including demographics, family background, educational and economic aspirations, and performance and behavior in school. Additionally, it asked about participation in a range of school activities, including football and several other school sports. Our initial pool of ``treated'' subjects were those men who indicated that they participated in or intended to participate in school football.

Before proceeding, it is critical to stress that the Add Health dataset does not contain a direct measure of actual football participation or exposure to football-related head trauma. This means that our treated group may in fact contain some non-compliers who, despite indicating that they intended to participate in football, ultimately did not. This may limit the applicability and generalizability of our eventual results.

Since our main interest is in the effects of playing football, we removed the 993 men in the core sample who participated in or intended to participate in at least one of field hockey, ice hockey, soccer and wrestling, as these sports are all associated with a high risk of repetitive head trauma.

Included in these 993 excluded men were those who participated in ``Other sports.'' Supplemental Table 1 summarizes how many of the initial pool of 3,989 men played each sport.

As part of the Wave I in-home interview conducted in 1995, subjects were asked several questions related to physical or functional limitations. Using a subset of these questions (listed in Supplemental Table 2), we excluded an additional 119 men who we determined had a physical or functional disability or limitation. Finally, 680 (140 football players, 540 controls) of the remaining 2,877 men were missing measurements of the primary outcome, the CES-D score measured in the 2008 in-home survey (Wave IV).

Following these exclusions, we are left with 2,197 subjects, of whom 521 indicated that they participated in or intended to participate in football. Of the 1,676 control subjects, 610 played a non-collision sport like basketball, swimming, or tennis, and the remaining 1,066 did not participate in any school sport.

**Supplemental Statistical Details**

**Details on Matching**

A common strategy in observational studies is to create matched sets consisting of treated and control subjects who are comparable. Ideally, within a matched set, treated and control subjects would be identical along these baseline variables. With several baseline variables, however, it is typically impossible to achieve this goal with any reasonable sample size. Instead, we aim to create matched sets which balance the distributions of each of the baseline variables between treated and control groups. To assess the suitability of a match, we look at the standardized difference in the mean of each covariate between the treated and control groups.

To construct balanced matched sets, we began by estimating the *propensity score*, the conditional probability of being assigned treatment given the baseline covariates. As [3] shows, the treatment assignment and covariates are conditionally independent given the propensity score so matching based on the propensity score tends to balance the distribution of the observed covariates between treated and control groups. Rather than matching strictly on the estimated propensity score, we instead match based on propensity score-calipered rank-based Mahalanobis distance between the observed covariates of each pair of treated and control subject. The combination of a propensity caliper and Mahalanobis distance strives to achieve a good compromise between overall covariate balance and closeness of the covariates of matched subjects[4].

We divided our subjects into three groups, based on what grade they were in when they completed the in-school questionnaire. Specifically, we grouped seventh and eighth graders together, ninth and tenth graders together, and eleventh and twelfth graders together. Such stratification ensures that we do not match, for instance, a seventh-grade student with a twelfth-grader. Within each stratum, we performed variable ratio matching [5,6] with our calipered distance matrix.

We note that some subjects are missing measurements of certain baseline variables. To deal with missing data in matched observational studies, we follow the recommendation of [7]: for each baseline variable we create an indicator variable of missingness. We include these indicators in the matching algorithm along with the baseline covariates. See Chapter 9.4 of [8] for further details.

The variable ratio matching procedure employed within each grade-level stratum works as follows. We take K = 15 to be the maximum number of controls that we will allow to be matched to a single treated subject. Then we define sets $S_{1}$ = (1/3, 1], $S_{15}$ = [0, 1/16], and $S_{k}$ = (1/(k+2), 1/(k+1)) for k = 2, …, 14. For each k = 1, …, 15, we select all subjects whose estimated propensity scores fall into the interval $S_{k}$ and perform 1:k-matching among these selected subjects. Whenever there are more than k times as many control subjects as treated subjects in one of these intervals, some control subjects are dropped from the analysis. Additionally, when there are more treated subjects than control subjects in the selected set, we build pair matches, optimally discarding the extra treated subjects who are most dissimilar to the controls within the stratum under consideration [9]. We note that our matching procedure does not use the full set of study subjects; instead it attempts to optimally discard subjects for whom there are comparable subjects with the opposite treatment assignment.

Following the example of [10], we considered several propensity score estimates until we were able to adequately balanced our football and control groups. For further details, see our pre-analysis protocol. We found that there were some football players with lower propensity score than all controls. We also found that there were some controls with larger propensity scores than football players. These are examples of individuals that lack common support; essentially for these individuals, there are no subjects with the opposition treatment status but similar propensity score and covariates. As a result, we dropped these subjects before running our matching algorithm.

Further details on how we estimated the propensity score are available in our pre-analysis protocol.

**Outcome Analysis**

To test the sharp null hypothesis that the effect of playing football was equal to some constant $\tau_{0}$, we first subtract $\tau_{0}$ from the treated subjects’ outcome, aligned the covariates within matched sets, and fit a BART regression model to predict the aligned adjusted responses. We then perform a permutational t-test on the residuals at the $\alpha=0.05$ level. Inverting these tests over a range of $\tau_{0}$ values yields an interval estimate for the effect of playing football. We further report the value of $\tau_{0}$ which maximizes the corresponding p-value as a point estimate of this effect.

**Ordered Hypothesis Testing**

Students who participate in high school sports may differ substantially from non-participants in terms of personality, temperament, and overall fitness and lifestyle (all of which are unmeasured). The sport control group is arguably a closer and more appropriate control group for our study and including non-sport controls may introduce problematic unmeasured confounding. However, simply dropping the non-sport controls from our analysis would cut our effective sample size by about 1/3 and may result in a substantial decrease in power. By constructing comparisons between the treated group and all controls as well as both control groups separately we systematically vary the unmeasured confounders of concern. Agreement across these three comparisons provide evidence that an ostensible treatment effect is a result of playing football and not due to unmeasured differences in students who played sports in high school and those who did not. Equivalence between the non-collision sport and non-sport control groups would provide further evidence that we are indeed testing for the effect of playing high school football.

In order to preserve the increased power of using controls from both groups while still testing the treated group against each group separately we follow an ordered testing procedure which controls the family wise error rate (FWER) [11] In particular, we first test the null of no treatment effect using matched sets constructed with all controls. If we reject that at level α, we conduct the same test separately using matched sets constructed using non collision-sport controls and non-sport controls. If we reject both separate tests at level α, we perform an equivalence test between the two control groups. If at any stage of the ordered testing procedure we do not reject, we stop the procedure. For example, if we do not reject the test using all controls we do not continue on to test against the two control groups separately. This stopping rule guarantees FWER control at level α.

In our main analysis, when testing the hypothesis of no treatment effect, we never reached comparisons 2 – 4 in this procedure. For the sake of completeness, we also the report the marginal 95% confidence intervals that go along with each test, regardless of whether we actually reached the test in the ordered testing procedure.

**Availability of data and analysis code**

This research uses data from Add Health, a program project designed by J. Richard Udry, Peter S. Bearman, and Kathleen Mullan Harris, and funded by a grant P01-HD31921 from the Eunice Kennedy Shriver National Institute of Child Health and Human Development, with cooperating funding from 17 other agencies. Special acknowledgement is due Ronal R. Rindfuss and Barbara Entwisle for assistance in the original design. Persons interested in obtaining Data Files from Add Health should contact Add Health, The University of North Carolina at Chapel Hill, Carolina Population Center, 206 W. Franklin Street, Chapel Hill, NC 27516-2524 (addhealth_contracts@unc.edu). No direct support was received from grant P01-HD31921 for this analysis.

All of our analysis was conducted in R. All analysis code is available on request from the first author.

**Baseline variables**

The corresponding Add Health variable names are included in brackets.

*Demographic Data*: age, height in 1994-95, weight in 1994-95, self-identified race [H1GI6A – H1GI6E], whether subject was born in the US [H1GH11], whether subject lived in a rural, suburban, or rural environment [H1IR12]

*Family Background*: how far mother/father went in school [H1RM1/H1RF1], whether mother/father was born in the US [H1RM2/H1RM2], type of work done by mother/father [H1RM4/H1RF4], whether mother/father works for pay [H1RM5/H1RF5], whether mother/father worked for pay in past year [H1RM6/H1RF6], whether mother/father received public assistance [H1RM9/H1RF9], whether mother/father smoked cigarettes [H1RF14/H1RF14],

*Relationship to mother/father:* how close subject felt to mother/father [H1WP9/H1WP13], how much subject thinks mother/father cared for him [H1WP10/H1WP14], whether subject felt mother / father was warm and loving towards him [H1PF1/H1PF23], whether subject’s mother encouraged him to be independent [H1PF2], whether subject’s mother provided constructive feedback when he had done wrong [H1PF3], whether subject is satisfied with communication with mother/father [H1PF4/H1PF24], whether subject is overall satisfied with relationship with mother / father [H1PF5/ H1PF25]

*School Performance and Academic Experience*: whether subject was in school at time of interview [H1GI18], current or most recent grade in school [H1GI20], most recent grade in English/ math/ history or social sciences / science [H1ED11 – H1ED14], frequency with which subject had trouble getting along with teachers [H1ED15], frequency with which subject had trouble paying attention in school [H1ED16], frequency with which subject had trouble complete homework assignments [H1ED17].

*General Health*: number of times subject exercised in past week [H1DA6], self-report of general health [H1GH1], number of times subject had routine physical exam [H1HS1], psychological counseling [H1HS3], or attended druge or alcohol abuse treatment program [H1HS5].

*Frequency of following conditions in past year:* headache [H1GH2], feeling hot all over suddenly [H1GH3], stomachache [H1GH4], cold sweats [H1GH5], physical weakness [H1GH6], sore throat or cough [H1GH7], tiredness [H1GH8], painful or frequent urination [H1GH9], feeling very sick [H1GH10], waking up feeling tired [H1GH11], skin problems such as itching or pimples [H1GH12], dizziness [H1GH13], chest pains [H1GH14], muscle or joint aches [H1GH15], poor appetite [H1GH17], trouble sleeping [H1GH18], trouble relaxing [H1GH19]

*Propensity for “risky” behavior:* frequency with which subject wore helmet while riding bicycle [H1GH39] or motorcycle [H1GH31], frequency with which subject rode motorcycle [H1GH40], frequency with which subject wore seatbelt [H1GH42], frequency with which subject drove after drinking alcohol [H1GH43]

*Propensity for delinquent behavior:* We created a single measure of delinquency by counting the following activities in which the subject engaged at least once in the previous year

Painted graffiti [H1DS1], deliberately damaged property [H1DS2], lied to parents or guardians [H1DS3], shoplifted [H1DS4], got into a serious physical fight [H1DS5], hurt someone badly enough to need bandages [H1DS6], run away from home [H1DS7], drove a car without owner’s permission [H1DS8], stole something worth > $50 [H1DS9], went into building to steal something [H1DS10], used or threatened to use a weapon to get something from someone [H1DS11], sold marijuana or other drugs [H1DS12], stole something worth < $50 [H1DS13], took part in large group fight [H1DS14], was loud, rowdy, or unruly in public [H1DS15]

*Substance Abuse*:

Cigarettes: whether subject ever tried cigarette smoking [H1TO1], age when subject smoked first whole cigarette [H1TO2], whether subject ever smoked regulary [H1TO3], age when subject began smoking regularly [H1TO4], how often subject smoked [H1TO5], daily cigarette consumption [H1TO7], whether subject attempted to quit smoking in past six months [H1TO8]

Chewing tobacco: how often subject has chewed tobacco [H1TO10], age when subject first started chewing tobacco [H1TO11]

Alcohol: whether subject drank alcohol 2+ times [H1TO12], whether subject drank without parents or family [H1TO13], age when subject began drinking without family [H1TO14], how often subject drank [H1TO15], typical alcohol consumption [H1TO16], how often subject had 5+ drinks in a row [H1TO17], number of times subject has gotten very drunk [H1TO17]

Marijuana: age when subject first tried marijuana [H1TO30], number of times subject has used marijuana in lifetime [H1TO31], number of times subject used marijuana in past month [H1To32]

Other drugs: age when subject first tried cocaine/inhalants/other illicit drug [H1TO34 /H1TO37/H1TO40], number of times subject used cocaine/inhalants/other illicit drug in lifetime [H1TO35/H1TO38/H1TO41], number of times subject used cocaine/inhalants/other illicit drug in past month [H1TO36/H1TO39/H1TO42]

*Exposure to violence*: how often in past year subject saw someone shot or stabbed in past year [H1FV1], someone pulled a knife or gun on subject [H1FV2], was shot [H1FV3], was cut or stabbed [H1FV4], got into a physical fight [H1FV5], was jumped [H1FV6], pulled a gun or knife on someone [H1FV7], shot or stabbed someone [H1FV8], carried a weapon to school [H1FV9]

*Suicidality*: whether subject seriously thought about committing suicide in past year [H1SU1], how many times did subject attempt suicide in past year [H1SU2], whether any of subjects’ friends or family members attempted suicide in past year [H1SU4 / H1SU6]

*Protective Factor*: how much did subject feel that adults, teachers, his parents, and friends cared about him [H1PR1 – H1PR4], people in his family understand him [H1PR5], wants to leave home [H1PR6], has fun with his family [H1PR7], his family pay attention to him [H1PR8]

*Feeling*: subjects were asked how often each of the following statements were true during the past week.

Bothered by things that usually don’t bother him [H1FS1], poor appetite [H1FS2], felt that he could not shake off the blues [H1FS3], felt that he was just as good as other people [H1FS4], had trouble keeping his mind on what he was doing [H1FS5], felt depressed [H1FS6], felt too tired to do things [H1FS7], felt hopeful about the future [H1FS8], thought his life had been a failure [H1FS9], felt fearful [H1FS10], felt happy [H1FS11], talked less than usual [H1FS12], felt lonely [H1FS13], felt people were unfriendly to him [H1FS14], enjoyed life [H1FS15], felt sad [H1FS16], felt that people disliked him [H1FS17], felt it was hard to get started doing things [H1FS18], felt life was not worth living [H1FS19]

*Personality*: Subjects were asked how often they agreed with the following statements

He never argues with anyone [H1PF7], when he gets what he wants, it’s because he worked hard [H1PF8], he never gets sad [H1PF10], he never criticizes other people [H1PF13], he usually goes out of his way to avoid dealing with problems [H1PF14], is upset by difficult problems [H1PF15], goes with his “gut feeling” without thinking much when making decisions [H1PF16], when he has a problem to solve, he gets as many facts as possible [H1PF18], when he has a problem to solve, he tries to think of as many different approaches as possible [H1PF19], when making decisions, he systematically compares alternatives [H1PF20], after solving a problem, he analyzes went right and wrong [H1FP21], he has lots of energy [H1PF26], seldom gets sick [H1PF27], when he gets sick, he gets better quickly [H1PF28], he is well coordinated [H1PF29], he has many good qualities [H1PF30], he is physically fit [H1PF31], he has a lot to be proud of [H1PF32], he likes himself just the way he is [H1PF33], he feels like he is doing everything just about right [H1PF34], he feels socially accepted [H1PF35], he feels loved and wanted [H1PF36]

*Expectations, Employment, and Income*: how much subject wants to go to college [H1EE1], how likely it is that subject will go to college [H1EE2], how many hours subject works for pay in non-summer week [H1EE4]

*Variables from the in-school questionnaire:* how often subject smoked, drank, or got drunk in past year [S59A – S59C], how often subject did something dangerous on a dare in past year [S59E], how often subject lied to parent or guardian [S59F], how often subject skipped school without excuse [S59G], how many times in a normal week does subject work, play, or exercise hard enough to sweat and breathe heavily [S63], whether he felt close to people at school [S62B], like he is part of the school [S62C], that students at his school are prejudiced [S62G], that he is happy to be at his school [S62I], that teachers at his school treat him fairly [S62L], that he feels safe in his neighborhood [S62Q] and school [S62R].

**References**

1. Harris KM. The Add Health Study : Design and Accomplishments University of North Carolina at Chapel Hill. 2012.

2. Resnick MD, Bearman PS, Blum RW, Bauman KE, Harris KM, Jones J, et al. Protecting Adolescents from Harm. JAMA. 1997;278: 823–832.

3. Rosenbaum PR, Rubin DB. The central role of the propensity score in observational studies for causal effects. Biometrika. 1983;70: 41–55. doi:10.1017/CBO9780511810725.016

4. Gu XS, Rosenbaum PR. Comparison of Multivariate Matching Methods: Structures, Distances, and Algorithms. J Comput Graph Stat. 1993;2: 405–420. doi:10.2307/1390693

5. Ming K, Rosenbaum PR. Substantial Gains in Bias Reduction from Matching with a Variable Number of Controls. Biometrics. 2000;56: 118–124.

6. Pimentel SD, Yoon F, Keele L. Variable-ratio matching with fine balance in a study of the Peer Health Exchange. Stat Med. 2015;34: 4070–4082. doi:10.1002/sim.6593

7. Rosenbaum PR, Rubin DB. Reducing bias in observational studies using subclassification on the propensity score. J Am Stat Assoc. 1984;79: 516–524.

8. Rosenbaum PR. Observational Studies. New York: Spring; 2002.

9. Rosenbaum PR. Optimal Matching of an Optimally Chosen Subset in Observational Studies. J Comput Graph Stat. 2012;21: 57–71.

10. Dehejia RH, Wahba S. Causal Effects in Nonexperimental Studies: Reevaluating the Evaluation of Training. Source J Am Stat Assoc. 1999.

11. Rosenbaum PR. Testing Hypothesis in Order. Biometrika. 2008;95: 248–252. doi:10.1093/biomet/asm085

**Table A. Sport participation information.**

| Sport | Number of Participants (out of initial pool of 3,989) | Number of Participants among football players | Number of participants among controls |
| --- | --- | --- | --- |
| Baseball/softball | 877 | 177 | 255 |
| Basketball | 1145 | 231 | 362 |
| Field Hockey^a^ | 44 | 0 | 0 |
| Football | 1035 | 521 | 0 |
| Ice hockey^a^ | 136 | 0 | 0 |
| Soccer^a^ | 380 | 0 | 0 |
| Swimming | 172 | 16 | 39 |
| Tennis | 180 | 6 | 52 |
| Track | 495 | 119 | 101 |
| Volleyball | 112 | 12 | 24 |
| Wrestling^a^ | 310 | 0 | 0 |
| Other^a^ | 433 | 0 | 0 |

^a^ Men playing field hockey, ice hockey, soccer, wrestling and other sports were excluded.

**Table B. Summary of responses used to determine whether Add Health subject had a physical limitation or disability**.

| Variable^a^ | Count^b^ |
| --- | --- |
| Do you have difficulty using your hands, arms, legs, or feet because of a permanent physical condition? [H1PL1] | 61 |
| Do you use a cane, crutches, walker, medically prescribed shoes, wheelchair, or scooter to get around because of a permanent physical condition [H1PL5] | 16 |
| Do you use a brace for your hand, arm, leg, or foot because of permanent physical condition [H1PL6] | 57 |
| Do you have an artificial hand, arm, leg, or foot? [H1PL7] | 3 |
| Do you have difficulty using your hands, arms, legs or feet because of physical condition that has lasted for the past twelve months or more? [H1GH1A] | 21 |

^a^ Add Health variable names are shown in brackets.

^b^ Counts are reported out of the 2,996 subjects who did not play a non-football sport with high incidence of head trauma. Subjects who answered yes on at least one of these questions were excluded from our analysis

**Table C. Composition of matched sets.**

|  | Comparison 1 (FB vs all controls) | Comparison 2 (FB vs sport controls) | Comparison 3 (FB vs non-sport controls) | Comparison 4 (sport controls vs non-sport controls) |
| --- | --- | --- | --- | --- |
| Composition of Matched Set^a^ (treated:control) |  |  |  |  |
| 1:1 | 290 | 264 | 342 | 426 |
| 1:2 | 47 | 31 | 45 | 87 |
| 1:3 | 49 | 16 | 23 | 33 |
| 1:4 | 9 | 1 | 20 | 9 |
| 1:5 | 17 | 5 | 0 | 0 |
| 1:6 | 10 | 2 | 3 | 1 |
| 1:7 | 8 | 0 | 0 | 0 |
| 1:8 | 2 | 0 | 1 | 0 |
| 1:9 | 3 | 0 | 0 | 0 |
| 1:10 | 1 | 0 | 0 | 0 |
| 1:11 | 2 | 0 | 0 | 0 |
| 1:12 | 0 | 0 | 0 | 0 |
| 1:13 | 0 | 0 | 0 | 0 |
| 1:14 | 0 | 0 | 0 | 0 |
| 1:15 | 17 | 0 | 0 | 0 |
| Total Matched Subjects (treated / control) | 1481 (447 / 1034) | 734 (415 / 319) | 1047 (437 / 610) | 1297 (556/741) |

^a^ In reporting the composition of a matched set, the left number is the number of “treated” subjects (i.e. football players in Comparisons 1 – 3 and sport controls in Comparison 4) to controls.

**Table D. Comparison of average baseline variables for football players vs all controls**.

|  | Before Matching | | After Matching | | Standardized Difference | |
| --- | --- | --- | --- | --- | --- | --- |
| Variable | Football | All Controls | Football | All Controls | Before | After |
| Age in 2008 (yrs) | 28.76 | 29.06 | 28.71 | 28.71 | -0.173 | 0 |
| Weight in 1994-95 (kg) | 72.611 | 68.04 | 71.53 | 70.44 | 0.263 | 0.063 |
| Height in 1994-95 (cm) | 173.96 | 173.93 | 173.63 | 173.38 | 0.002 | 0.024 |
| Self-reported race |  |  |  |  |  |  |
| White (%) | 66.03 (344/521) | 72.55 (1216/1676) | 67.79 (303/447) | 66.34 (742/1034) | -0.175 | 0.039 |
| Black (%) | 27.45 (143/521) | 17.72 (297/1676) | 25.5 (114/447) | 26.75 (212/1034) | 0.282 | -0.036 |
| Native American (%) | 2.69 (14/521) | 2.63 (44/1676) | 2.46 (11/447) | 2.32 (24/1034) | 0.005 | 0.011 |
| Asian (%) | 2.69 (14/521) | 4.83 (81/1676) | 2.46 (11/447) | 2.81 (42/1034) | -0.155 | -0.025 |
| Other (%) | 4.99 (26/521) | 6.26 (105/1676) | 4.92 (22/447) | 5.34 (49/1034) | -0.072 | -0.024 |
| Self-reported rating of general health |  |  |  |  |  |  |
| Excellent (%) | 39.35 (205/521) | 30.91 (518/1676) | 38.7 (173/447) | 37.59 (340/1034) | 0.22 | 0.029 |
| Very good (%) | 39.54 (206/521) | 39.56 (663/1676) | 39.82 (178/447) | 40.79 (425/1034) | 0 | -0.025 |
| Good (%) | 16.89 (88/521) | 24.58 (412/1676) | 17 (76/447) | 17.57 (227/1034) | -0.25 | -0.019 |
| Fair (%) | 3.84 (20/521) | 4.42 (74/1676) | 4.03 (18/447) | 3.74 (39/1034) | -0.037 | 0.018 |
| Poor(%) | 0.38 (2/521) | 0.48 (8/1676) | 0.45 (2/447) | 0.31 (3/1034) | -0.019 | 0.028 |
| How often in past week subject exercised |  |  |  |  |  |  |
| Not at all (%) | 15.55 (81/521) | 23.21 (389/1676) | 14.99 (67/447) | 15.82 (217/1034) | -0.257 | -0.028 |
| 1 - 2 times (%) | 28.21 (147/521) | 30.79 (516/1676) | 29.31 (131/447) | 28.99 (312/1034) | -0.072 | 0.009 |
| 3 - 4 times (%) | 23.99 (125/521) | 22.32 (374/1676) | 25.28 (113/447) | 26.68 (258/1034) | 0.05 | -0.041 |
| 5+ times (%) | 32.25 (168/521) | 23.63 (396/1676) | 30.43 (136/447) | 28.52 (247/1034) | 0.236 | 0.052 |
| Not at all (%) | 15.55 (81/521) | 23.21 (389/1676) | 14.99 (67/447) | 15.82 (217/1034) | -0.257 | -0.028 |
| How often in past year has subject had |  |  |  |  |  |  |
| Headaches |  |  |  |  |  |  |
| Never (%) | 10.17 (53/521) | 11.52 (193/1676) | 10.07 (45/447) | 11.5 (116/1034) | -0.055 | -0.059 |
| A few times (%) | 70.44 (367/521) | 67.18 (1126/1676) | 70.25 (314/447) | 66.23 (702/1034) | 0.089 | 0.11 |
| Once a week (%) | 16.51 (86/521) | 17.6 (295/1676) | 17 (76/447) | 20.54 (187/1034) | -0.037 | -0.119 |
| Almost every day (%) | 2.5 (13/521) | 2.98 (50/1676) | 2.24 (10/447) | 1.64 (26/1034) | -0.039 | 0.047 |
| Every day (%) | 0.38 (2/521) | 0.66 (11/1676) | 0.45 (2/447) | 0.1 (3/1034) | -0.052 | 0.067 |
| Unexplained Physical Weakness |  |  |  |  |  |  |
| Never (%) | 61.23 (319/521) | 60.44 (1013/1676) | 61.3 (274/447) | 63.41 (661/1034) | 0.02 | -0.054 |
| A few times (%) | 34.74 (181/521) | 33.59 (563/1676) | 34.68 (155/447) | 33.26 (332/1034) | 0.03 | 0.037 |
| Once a week (%) | 3.84 (20/521) | 4.65 (78/1676) | 3.8 (17/447) | 2.63 (34/1034) | -0.052 | 0.075 |
| Almost every day (%) | 0.19 (1/521) | 0.95 (16/1676) | 0.22 (1/447) | 0.66 (6/1034) | -0.162 | -0.092 |
| Every day (%) | 0 (0/521) | 0.3 (5/1676) | 0 (0/447) | 0.04 (1/1034) | -0.15 | -0.022 |
| Dizziness |  |  |  |  |  |  |
| Never (%) | 69.1 (360/521) | 65.21 (1093/1676) | 68.9 (308/447) | 68.93 (704/1034) | 0.105 | -0.001 |
| A few times (%) | 25.53 (133/521) | 29.47 (494/1676) | 27.07 (121/447) | 28.26 (294/1034) | -0.113 | -0.034 |
| Once a week (%) | 4.22 (22/521) | 3.64 (61/1676) | 3.58 (16/447) | 2.09 (25/1034) | 0.037 | 0.094 |
| Almost every day (%) | 0.77 (4/521) | 1.01 (17/1676) | 0.45 (2/447) | 0.46 (8/1034) | -0.034 | -0.002 |
| Every day (%) | 0.38 (2/521) | 0.6 (10/1676) | 0 (0/447) | 0.25 (3/1034) | -0.041 | -0.049 |
| Muscle or joint pain |  |  |  |  |  |  |
| Never (%) | 11.13 (58/521) | 20.17 (338/1676) | 10.96 (49/447) | 13.57 (181/1034) | -0.34 | -0.098 |
| A few times (%) | 50.86 (265/521) | 53.34 (894/1676) | 52.35 (234/447) | 53.37 (585/1034) | -0.062 | -0.026 |
| Once a week (%) | 28.6 (149/521) | 20.41 (342/1676) | 28.86 (129/447) | 24.26 (207/1034) | 0.233 | 0.131 |
| Almost every day (%) | 7.29 (38/521) | 5.01 (84/1676) | 6.94 (31/447) | 7.61 (53/1034) | 0.114 | -0.034 |
| Every day (%) | 2.11 (11/521) | 1.01 (17/1676) | 0.89 (4/447) | 1.19 (8/1034) | 0.102 | -0.028 |
| Trouble Sleeping |  |  |  |  |  |  |
| Never (%) | 44.15 (230/521) | 42.96 (720/1676) | 44.3 (198/447) | 44.13 (449/1034) | 0.03 | 0.004 |
| A few times (%) | 37.62 (196/521) | 34.67 (581/1676) | 38.93 (174/447) | 37.83 (392/1034) | 0.077 | 0.029 |
| Once a week (%) | 11.32 (59/521) | 12.83 (215/1676) | 11.41 (51/447) | 11.35 (118/1034) | -0.059 | 0.002 |
| Almost every day (%) | 4.61 (24/521) | 7.04 (118/1676) | 3.8 (17/447) | 5.63 (57/1034) | -0.139 | -0.104 |
| Every day (%) | 2.3 (12/521) | 2.45 (41/1676) | 1.57 (7/447) | 1.06 (18/1034) | -0.012 | 0.042 |
| Seriously contemplated suicide (%) | 9.4 (49/521) | 10.2 (171/1676) | 7.38 (33/447) | 6.49 (78/1034) | -0.034 | 0.038 |
| Desire to go to college |  |  |  |  |  |  |
| 1 - low (%) | 3.26 (17/521) | 5.43 (91/1676) | 2.68 (12/447) | 2.5 (33/1034) | -0.144 | 0.012 |
| 2 (%) | 1.34 (7/521) | 4.18 (70/1676) | 1.34 (6/447) | 2.3 (31/1034) | -0.259 | -0.087 |
| 3 - medium (%) | 7.49 (39/521) | 12.17 (204/1676) | 7.38 (33/447) | 8.09 (108/1034) | -0.212 | -0.032 |
| 4 (%) | 14.01 (73/521) | 15.63 (262/1676) | 14.77 (66/447) | 14.42 (158/1034) | -0.058 | 0.012 |
| 5 - high (%) | 73.7 (384/521) | 62.35 (1045/1676) | 73.6 (329/447) | 72.59 (703/1034) | 0.317 | 0.028 |
| Likelihood to go to college (%) |  |  |  |  |  |  |
| 1 - low (%) | 3.84 (20/521) | 6.38 (107/1676) | 3.13 (14/447) | 2.98 (37/1034) | -0.157 | 0.009 |
| 2 (%) | 4.03 (21/521) | 6.38 (107/1676) | 4.47 (20/447) | 4.12 (55/1034) | -0.143 | 0.021 |
| 3 - medium (%) | 14.2 (74/521) | 16.17 (271/1676) | 13.2 (59/447) | 13.29 (138/1034) | -0.07 | -0.003 |
| 4 (%) | 23.99 (125/521) | 23.57 (395/1676) | 23.94 (107/447) | 24.76 (268/1034) | 0.013 | -0.024 |
| 5 - high (%) | 53.74 (280/521) | 47.2 (791/1676) | 55.03 (246/447) | 54.74 (535/1034) | 0.165 | 0.007 |
| Ever tried cigarette smoking (%) | 53.55 (279/521) | 56.09 (940/1676) | 51.23 (229/447) | 49.65 (508/1034) | -0.064 | 0.04 |
| Smoked regularly (%) | 14.01 (73/521) | 20.47 (343/1676) | 12.08 (54/447) | 12 (135/1034) | -0.226 | 0.003 |
| Ever drank alcohol (%) | 55.09 (287/521) | 55.01 (922/1676) | 53.02 (237/447) | 49.72 (484/1034) | 0.002 | 0.083 |
| How often subject drank alcohol in past year |  |  |  |  |  |  |
| Almost every day (%) | 0.77 (4/521) | 1.19 (20/1676) | 0.45 (2/447) | 1.43 (8/1034) | -0.058 | -0.134 |
| 3 - 5 days/week (%) | 2.88 (15/521) | 3.28 (55/1676) | 2.24 (10/447) | 2.48 (20/1034) | -0.03 | -0.018 |
| 1 - 2 days/week (%) | 6.33 (33/521) | 6.86 (115/1676) | 5.82 (26/447) | 5.52 (52/1034) | -0.027 | 0.015 |
| 2 - 3 days/month (%) | 10.17 (53/521) | 7.1 (119/1676) | 10.29 (46/447) | 7.4 (64/1034) | 0.132 | 0.124 |
| Once a month or less (%) | 10.56 (55/521) | 11.1 (186/1676) | 10.07 (45/447) | 9.17 (103/1034) | -0.022 | 0.036 |
| Once or twice in past year (%) | 14.4 (75/521) | 14.98 (251/1676) | 14.09 (63/447) | 14.41 (141/1034) | -0.021 | -0.011 |
| Never (%) | 9.79 (51/521) | 10.14 (170/1676) | 10.07 (45/447) | 9.31 (96/1034) | -0.015 | 0.032 |
| Not applicable (%) | 45.11 (235/521) | 45.35 (760/1676) | 46.98 (210/447) | 50.28 (550/1034) | -0.005 | -0.066 |
| How often in past year subject drank 5+ alcoholic drinks in a row |  |  |  |  |  |  |
| Almost every day (%) | 0.77 (4/521) | 0.72 (12/1676) | 0.45 (2/447) | 0.25 (2/1034) | 0.008 | 0.029 |
| 3 - 5 days/week (%) | 1.73 (9/521) | 2.39 (40/1676) | 1.12 (5/447) | 2 (13/1034) | -0.061 | -0.082 |
| 1 - 2 days/week (%) | 4.99 (26/521) | 4.95 (83/1676) | 4.92 (22/447) | 4.61 (37/1034) | 0.002 | 0.018 |
| 2 - 3 days/month (%) | 6.91 (36/521) | 5.31 (89/1676) | 6.94 (31/447) | 4.92 (45/1034) | 0.081 | 0.102 |
| Once a month or less (%) | 6.14 (32/521) | 5.61 (94/1676) | 5.82 (26/447) | 5.87 (48/1034) | 0.028 | -0.003 |
| Once or twice in past year (%) | 7.29 (38/521) | 8.41 (141/1676) | 6.94 (31/447) | 6.84 (76/1034) | -0.053 | 0.004 |
| Never (%) | 16.89 (88/521) | 17.06 (286/1676) | 16.33 (73/447) | 15.93 (167/1034) | -0.006 | 0.014 |
| Not applicable (%) | 55.28 (288/521) | 55.55 (931/1676) | 57.49 (257/447) | 59.58 (646/1034) | -0.005 | -0.042 |
| How much subject agrees that |  |  |  |  |  |  |
| You never get sad |  |  |  |  |  |  |
| Strongly agree (%) | 4.03 (21/521) | 5.37 (90/1676) | 4.03 (18/447) | 5.66 (55/1034) | -0.083 | -0.101 |
| Agree (%) | 15.16 (79/521) | 13.9 (233/1676) | 15.21 (68/447) | 15.12 (151/1034) | 0.045 | 0.003 |
| Neither agree nor disagree (%) | 19.77 (103/521) | 20.88 (350/1676) | 19.02 (85/447) | 18.35 (206/1034) | -0.035 | 0.021 |
| Disagree (%) | 53.17 (277/521) | 49.7 (833/1676) | 54.14 (242/447) | 50.88 (528/1034) | 0.087 | 0.082 |
| Strongly disagree (%) | 7.87 (41/521) | 10.08 (169/1676) | 7.61 (34/447) | 9.98 (94/1034) | -0.101 | -0.108 |
| You are physically fit |  |  |  |  |  |  |
| Strongly agree (%) | 42.61 (222/521) | 31.38 (526/1676) | 40.94 (183/447) | 43.1 (353/1034) | 0.289 | -0.056 |
| Agree (%) | 47.6 (248/521) | 47.49 (796/1676) | 48.77 (218/447) | 45.28 (509/1034) | 0.003 | 0.088 |
| Neither agree nor disagree (%) | 8.06 (42/521) | 14.26 (239/1676) | 8.28 (37/447) | 9.02 (118/1034) | -0.269 | -0.032 |
| Disagree (%) | 1.73 (9/521) | 6.03 (101/1676) | 2.01 (9/447) | 2.51 (51/1034) | -0.34 | -0.039 |
| Strongly disagree (%) | 0 (0/521) | 0.72 (12/1676) | 0 (0/447) | 0.07 (3/1034) | -0.233 | -0.024 |
| You feel socially accepted |  |  |  |  |  |  |
| Strongly agree (%) | 38.2 (199/521) | 31.8 (533/1676) | 36.24 (162/447) | 38.89 (355/1034) | 0.167 | -0.069 |
| Agree (%) | 54.13 (282/521) | 54.47 (913/1676) | 56.15 (251/447) | 51.99 (568/1034) | -0.009 | 0.105 |
| Neither agree nor disagree (%) | 6.14 (32/521) | 9.61 (161/1676) | 6.26 (28/447) | 7.13 (81/1034) | -0.173 | -0.043 |
| Disagree (%) | 1.15 (6/521) | 3.76 (63/1676) | 0.89 (4/447) | 1.76 (28/1034) | -0.255 | -0.084 |
| Strongly disagree (%) | 0.38 (2/521) | 0.24 (4/1676) | 0.45 (2/447) | 0.24 (2/1034) | 0.031 | 0.044 |
| How much subject feels his parents care about him |  |  |  |  |  |  |
| Not at all (%) | 0.19 (1/521) | 0.36 (6/1676) | 0 (0/447) | 0.11 (1/1034) | -0.044 | -0.03 |
| Very little (%) | 0.77 (4/521) | 0.6 (10/1676) | 0.89 (4/447) | 0.6 (5/1034) | 0.025 | 0.043 |
| Somewhat (%) | 2.3 (12/521) | 2.63 (44/1676) | 1.57 (7/447) | 2.15 (27/1034) | -0.027 | -0.048 |
| Quite a bit (%) | 10.56 (55/521) | 12.95 (217/1676) | 9.62 (43/447) | 9.96 (121/1034) | -0.096 | -0.014 |
| Very much (%) | 85.99 (448/521) | 83.17 (1394/1676) | 87.7 (392/447) | 86.94 (878/1034) | 0.1 | 0.027 |

**Table E. Comparison of average baseline variables for football players vs sport controls.**

|  | Before Matching | | After Matching | | Standardized Difference | |
| --- | --- | --- | --- | --- | --- | --- |
|  | Football | Sport controls | Football | Sport controls | Before | After |
|  |  |  |  |  |  |  |
| Age in 2008 (yrs) | 28.76 | 28.94 | 28.66 | 28.66 | -0.109 | 0 |
| Weight in 1994-95 (kg) | 72.611 | 67.88 | 70.54 | 68.89 | 0.29 | 0.101 |
| Height in 1994-95 (cm) | 173.96 | 175.59 | 173.76 | 174.14 | -0.157 | -0.038 |
| Self-reported race |  |  |  |  |  |  |
| White (%) | 66.03 (344/521) | 71.64 (437/610) | 68.34 (218/319) | 67.99 (290/415) | -0.138 | 0.009 |
| Black (%) | 27.45 (143/521) | 21.97 (134/610) | 27.27 (87/319) | 25.77 (97/415) | 0.144 | 0.04 |
| Native American (%) | 2.69 (14/521) | 1.8 (11/610) | 2.19 (7/319) | 2.31 (10/415) | 0.067 | -0.009 |
| Asian (%) | 2.69 (14/521) | 5.74 (35/610) | 2.19 (7/319) | 4.1 (22/415) | -0.185 | -0.115 |
| Other (%) | 4.99 (26/521) | 3.44 (21/610) | 2.51 (8/319) | 3.87 (13/415) | 0.086 | -0.076 |
| Self-reported rating of general health |  |  |  |  |  |  |
| Excellent (%) | 39.35 (205/521) | 37.05 (226/610) | 41.07 (131/319) | 38.15 (159/415) | 0.054 | 0.069 |
| Very good (%) | 39.54 (206/521) | 42.3 (258/610) | 40.44 (129/319) | 45.05 (183/415) | -0.064 | -0.108 |
| Good (%) | 16.89 (88/521) | 17.21 (105/610) | 15.36 (49/319) | 14.03 (63/415) | -0.01 | 0.041 |
| Fair (%) | 3.84 (20/521) | 2.95 (18/610) | 2.82 (9/319) | 2.46 (9/415) | 0.055 | 0.023 |
| Poor(%) | 0.38 (2/521) | 0.33 (2/610) | 0.31 (1/319) | 0.31 (1/415) | 0.011 | 0 |
| How often in past week subject exercised |  |  |  |  |  |  |
| Not at all (%) | 15.55 (81/521) | 20.33 (124/610) | 13.17 (42/319) | 17.36 (77/415) | -0.146 | -0.128 |
| 1 - 2 times (%) | 28.21 (147/521) | 28.52 (174/610) | 29.15 (93/319) | 27.78 (115/415) | -0.008 | 0.035 |
| 3 - 4 times (%) | 23.99 (125/521) | 21.48 (131/610) | 26.96 (86/319) | 22.58 (92/415) | 0.069 | 0.119 |
| 5+ times (%) | 32.25 (168/521) | 29.51 (180/610) | 30.72 (98/319) | 32.27 (131/415) | 0.068 | -0.038 |
| How often in past year has subject had |  |  |  |  |  |  |
| Headaches |  |  |  |  |  |  |
| Never (%) | 10.17 (53/521) | 11.64 (71/610) | 10.97 (35/319) | 12.79 (53/415) | -0.055 | -0.068 |
| A few times (%) | 70.44 (367/521) | 65.9 (402/610) | 70.85 (226/319) | 63.56 (266/415) | 0.113 | 0.181 |
| Once a week (%) | 16.51 (86/521) | 20.16 (123/610) | 15.99 (51/319) | 22.03 (89/415) | -0.11 | -0.181 |
| Almost every day (%) | 2.5 (13/521) | 1.8 (11/610) | 1.88 (6/319) | 1.31 (6/415) | 0.053 | 0.044 |
| Every day (%) | 0.38 (2/521) | 0.33 (2/610) | 0.31 (1/319) | 0.31 (1/415) | 0.011 | 0 |
| Unexplained Physical Weakness |  |  |  |  |  |  |
| Never (%) | 61.23 (319/521) | 62.62 (382/610) | 63.32 (202/319) | 62.08 (256/415) | -0.033 | 0.029 |
| A few times (%) | 34.74 (181/521) | 31.8 (194/610) | 33.86 (108/319) | 33.92 (143/415) | 0.071 | -0.002 |
| Once a week (%) | 3.84 (20/521) | 4.43 (27/610) | 2.82 (9/319) | 2.95 (12/415) | -0.034 | -0.008 |
| Almost every day (%) | 0.19 (1/521) | 0.49 (3/610) | 0 (0/319) | 0.94 (3/415) | -0.063 | -0.199 |
| Every day (%) | 0 (0/521) | 0.49 (3/610) | 0 (0/319) | 0.1 (1/415) | -0.138 | -0.029 |
| Dizziness |  |  |  |  |  |  |
| Never (%) | 69.1 (360/521) | 64.92 (396/610) | 66.77 (213/319) | 68.75 (282/415) | 0.103 | -0.048 |
| A few times (%) | 25.53 (133/521) | 30.98 (189/610) | 29.47 (94/319) | 28.92 (122/415) | -0.141 | 0.014 |
| Once a week (%) | 4.22 (22/521) | 2.62 (16/610) | 3.76 (12/319) | 1.39 (8/415) | 0.098 | 0.144 |
| Almost every day (%) | 0.77 (4/521) | 0.98 (6/610) | 0 (0/319) | 0.63 (2/415) | -0.027 | -0.079 |
| Every day (%) | 0.38 (2/521) | 0.33 (2/610) | 0 (0/319) | 0.31 (1/415) | 0.011 | -0.06 |
| Muscle or joint pain |  |  |  |  |  |  |
| Never (%) | 11.13 (58/521) | 15.57 (95/610) | 11.6 (37/319) | 14.02 (63/415) | -0.154 | -0.084 |
| A few times (%) | 50.86 (265/521) | 53.44 (326/610) | 51.72 (165/319) | 52.98 (225/415) | -0.059 | -0.029 |
| Once a week (%) | 28.6 (149/521) | 21.8 (133/610) | 30.72 (98/319) | 22.24 (88/415) | 0.178 | 0.222 |
| Almost every day (%) | 7.29 (38/521) | 7.87 (48/610) | 5.64 (18/319) | 9.82 (34/415) | -0.025 | -0.182 |
| Every day (%) | 2.11 (11/521) | 1.15 (7/610) | 0.31 (1/319) | 0.94 (5/415) | 0.084 | -0.054 |
| Trouble Sleeping |  |  |  |  |  |  |
| Never (%) | 44.15 (230/521) | 44.26 (270/610) | 45.77 (146/319) | 45.44 (186/415) | -0.003 | 0.007 |
| A few times (%) | 37.62 (196/521) | 35.74 (218/610) | 38.87 (124/319) | 36.82 (156/415) | 0.045 | 0.049 |
| Once a week (%) | 11.32 (59/521) | 11.97 (73/610) | 11.29 (36/319) | 10.59 (44/415) | -0.023 | 0.025 |
| Almost every day (%) | 4.61 (24/521) | 6.72 (41/610) | 2.82 (9/319) | 5.99 (24/415) | -0.108 | -0.162 |
| Every day (%) | 2.3 (12/521) | 1.15 (7/610) | 1.25 (4/319) | 1.15 (5/415) | 0.097 | 0.009 |
| Seriously contemplated suicide (%) | 9.4 (49/521) | 7.87 (48/610) | 4.7 (15/319) | 7.34 (28/415) | 0.062 | -0.106 |
| Desire to go to college |  |  |  |  |  |  |
| 1 - low (%) | 3.26 (17/521) | 2.95 (18/610) | 2.82 (9/319) | 2.35 (10/415) | 0.02 | 0.031 |
| 2 (%) | 1.34 (7/521) | 2.95 (18/610) | 1.57 (5/319) | 2.35 (8/415) | -0.135 | -0.066 |
| 3 - medium (%) | 7.49 (39/521) | 9.67 (59/610) | 5.96 (19/319) | 7.86 (33/415) | -0.091 | -0.079 |
| 4 (%) | 14.01 (73/521) | 11.64 (71/610) | 14.42 (46/319) | 11.19 (49/415) | 0.08 | 0.11 |
| 5 - high (%) | 73.7 (384/521) | 72.46 (442/610) | 75.24 (240/319) | 76.25 (315/415) | 0.032 | -0.026 |
| Likelihood to go to college (%) |  |  |  |  |  |  |
| 1 - low (%) | 3.84 (20/521) | 4.1 (25/610) | 2.19 (7/319) | 2.4 (11/415) | -0.015 | -0.012 |
| 2 (%) | 4.03 (21/521) | 4.43 (27/610) | 3.76 (12/319) | 4.25 (18/415) | -0.023 | -0.028 |
| 3 - medium (%) | 14.2 (74/521) | 11.31 (69/610) | 12.54 (40/319) | 9.81 (41/415) | 0.098 | 0.093 |
| 4 (%) | 23.99 (125/521) | 25.41 (155/610) | 25.08 (80/319) | 26.04 (105/415) | -0.038 | -0.026 |
| 5 - high (%) | 53.74 (280/521) | 54.26 (331/610) | 56.43 (180/319) | 57.49 (240/415) | -0.012 | -0.025 |
| Ever tried cigarette smoking (%) | 53.55 (279/521) | 53.28 (325/610) | 46.08 (147/319) | 48.21 (202/415) | 0.006 | -0.049 |
| Smoked regularly (%) | 14.01 (73/521) | 14.26 (87/610) | 7.52 (24/319) | 11.24 (49/415) | -0.008 | -0.123 |
| Ever drank alcohol (%) | 55.09 (287/521) | 54.26 (331/610) | 44.83 (143/319) | 49.95 (203/415) | 0.019 | -0.118 |
| How often subject drank alcohol in past year |  |  |  |  |  |  |
| Almost every day (%) | 0.77 (4/521) | 0.82 (5/610) | 0.31 (1/319) | 0.63 (2/415) | -0.007 | -0.041 |
| 3 - 5 days/week (%) | 2.88 (15/521) | 3.61 (22/610) | 0.63 (2/319) | 3.12 (15/415) | -0.048 | -0.164 |
| 1 - 2 days/week (%) | 6.33 (33/521) | 6.39 (39/610) | 4.7 (15/319) | 5.24 (19/415) | -0.003 | -0.025 |
| 2 - 3 days/month (%) | 10.17 (53/521) | 6.56 (40/610) | 8.78 (28/319) | 7.6 (29/415) | 0.146 | 0.047 |
| Once a month or less (%) | 10.56 (55/521) | 11.97 (73/610) | 8.15 (26/319) | 10.66 (44/415) | -0.052 | -0.092 |
| Once or twice in past year (%) | 14.4 (75/521) | 14.1 (86/610) | 11.91 (38/319) | 12.94 (53/415) | 0.01 | -0.034 |
| Never (%) | 9.79 (51/521) | 10.49 (64/610) | 10.03 (32/319) | 9.67 (40/415) | -0.027 | 0.014 |
| Not applicable (%) | 45.11 (235/521) | 46.07 (281/610) | 55.49 (177/319) | 50.15 (213/415) | -0.019 | 0.107 |
| How often in past year subject drank 5+ alcoholic drinks in a row |  |  |  |  |  |  |
| Almost every day (%) | 0.77 (4/521) | 0.49 (3/610) | 0 (0/319) | 0 (0/415) | 0.039 | 0 |
| 3 - 5 days/week (%) | 1.73 (9/521) | 2.46 (15/610) | 0.63 (2/319) | 2.47 (11/415) | -0.06 | -0.152 |
| 1 - 2 days/week (%) | 4.99 (26/521) | 5.41 (33/610) | 3.13 (10/319) | 4.99 (18/415) | -0.022 | -0.096 |
| 2 - 3 days/month (%) | 6.91 (36/521) | 3.61 (22/610) | 5.64 (18/319) | 2.8 (12/415) | 0.163 | 0.14 |
| Once a month or less (%) | 6.14 (32/521) | 6.23 (38/610) | 3.76 (12/319) | 5.55 (22/415) | -0.004 | -0.085 |
| Once or twice in past year (%) | 7.29 (38/521) | 9.51 (58/610) | 5.96 (19/319) | 8.87 (37/415) | -0.093 | -0.123 |
| Never (%) | 16.89 (88/521) | 15.57 (95/610) | 15.36 (49/319) | 15.19 (61/415) | 0.041 | 0.005 |
| Not applicable (%) | 55.28 (288/521) | 56.72 (346/610) | 65.52 (209/319) | 60.14 (254/415) | -0.03 | 0.111 |
| How much subject agrees that |  |  |  |  |  |  |
| You never get sad |  |  |  |  |  |  |
| Strongly agree (%) | 4.03 (21/521) | 4.92 (30/610) | 3.45 (11/319) | 4.68 (21/415) | -0.05 | -0.069 |
| Agree (%) | 15.16 (79/521) | 15.25 (93/610) | 15.99 (51/319) | 16.17 (67/415) | -0.003 | -0.006 |
| Neither agree nor disagree (%) | 19.77 (103/521) | 19.51 (119/610) | 18.5 (59/319) | 19.25 (78/415) | 0.008 | -0.022 |
| Disagree (%) | 53.17 (277/521) | 51.31 (313/610) | 55.8 (178/319) | 51.44 (216/415) | 0.043 | 0.1 |
| Strongly disagree (%) | 7.87 (41/521) | 8.85 (54/610) | 6.27 (20/319) | 8.46 (33/415) | -0.041 | -0.092 |
| You are physically fit |  |  |  |  |  |  |
| Strongly agree (%) | 42.61 (222/521) | 42.62 (260/610) | 40.75 (130/319) | 45.66 (186/415) | 0 | -0.114 |
| Agree (%) | 47.6 (248/521) | 45.41 (277/610) | 50.47 (161/319) | 43.83 (184/415) | 0.05 | 0.153 |
| Neither agree nor disagree (%) | 8.06 (42/521) | 9.02 (55/610) | 6.9 (22/319) | 8.73 (37/415) | -0.04 | -0.076 |
| Disagree (%) | 1.73 (9/521) | 2.46 (15/610) | 1.88 (6/319) | 1.47 (7/415) | -0.06 | 0.034 |
| Strongly disagree (%) | 0 (0/521) | 0.33 (2/610) | 0 (0/319) | 0.31 (1/415) | -0.112 | -0.107 |
| You feel socially accepted |  |  |  |  |  |  |
| Strongly agree (%) | 38.2 (199/521) | 36.72 (224/610) | 37.93 (121/319) | 39 (160/415) | 0.035 | -0.025 |
| Agree (%) | 54.13 (282/521) | 54.26 (331/610) | 56.11 (179/319) | 52.77 (221/415) | -0.003 | 0.077 |
| Neither agree nor disagree (%) | 6.14 (32/521) | 6.72 (41/610) | 5.96 (19/319) | 6.82 (27/415) | -0.027 | -0.041 |
| Disagree (%) | 1.15 (6/521) | 1.97 (12/610) | 0 (0/319) | 1.41 (7/415) | -0.079 | -0.136 |
| Strongly disagree (%) | 0.38 (2/521) | 0 (0/610) | 0 (0/319) | 0 (0/415) | 0.088 | 0 |
| How much subject feels his parents care about him |  |  |  |  |  |  |
| Not at all (%) | 0.19 (1/521) | 0.49 (3/610) | 0.31 (1/319) | 0.31 (1/415) | -0.063 | 0 |
| Very little (%) | 0.77 (4/521) | 0.49 (3/610) | 0.31 (1/319) | 0.63 (2/415) | 0.039 | -0.044 |
| Somewhat (%) | 2.3 (12/521) | 1.8 (11/610) | 1.25 (4/319) | 2.22 (9/415) | 0.04 | -0.077 |
| Quite a bit (%) | 10.56 (55/521) | 11.48 (70/610) | 10.97 (35/319) | 10.85 (44/415) | -0.034 | 0.004 |
| Very much (%) | 85.99 (448/521) | 85.41 (521/610) | 87.15 (278/319) | 85.99 (359/415) | 0.019 | 0.038 |

**Table F. Comparison of average baseline variables for football players vs non-sport controls.**

|  | Before Matching | | After Matching | | Standardized Difference | |
| --- | --- | --- | --- | --- | --- | --- |
|  | Football | Non-sport controls | Football | Non-sport controls | Before | After |
|  |  |  |  |  |  |  |
| Age in 2008 (yrs) | 28.76 | 29.13 | 28.6 | 28.63 | -0.207 | -0.015 |
| Weight in 1994-95 (kg) | 72.61 | 68.13 | 70.35 | 69.15 | 0.25 | 0.067 |
| Height in 1994-95 (cm) | 173.96 | 173 | 173 | 172.64 | 0.092 | 0.034 |
| Self-reported race |  |  |  |  |  |  |
| White (%) | 66.03 (344/521) | 73.08 (779/1066) | 66.59 (289/434) | 69.78 (441/607) | -0.181 | -0.082 |
| Black (%) | 27.45 (143/521) | 15.29 (163/1066) | 26.96 (117/434) | 23.72 (124/607) | 0.343 | 0.091 |
| Native American (%) | 2.69 (14/521) | 3.1 (33/1066) | 2.07 (9/434) | 2.08 (15/607) | -0.03 | -0.001 |
| Asian (%) | 2.69 (14/521) | 4.32 (46/1066) | 2.3 (10/434) | 2.32 (17/607) | -0.111 | -0.001 |
| Other (%) | 4.99 (26/521) | 7.88 (84/1066) | 5.07 (22/434) | 5.7 (32/607) | -0.147 | -0.032 |
| Self-reported rating of general health |  |  |  |  |  |  |
| Excellent (%) | 39.35 (205/521) | 27.39 (292/1066) | 38.02 (165/434) | 35.82 (198/607) | 0.299 | 0.055 |
| Very good (%) | 39.54 (206/521) | 37.99 (405/1066) | 41.71 (181/434) | 38.02 (235/607) | 0.038 | 0.09 |
| Good (%) | 16.89 (88/521) | 28.8 (307/1066) | 15.9 (69/434) | 21.48 (144/607) | -0.356 | -0.167 |
| Fair (%) | 3.84 (20/521) | 5.25 (56/1066) | 3.92 (17/434) | 4.09 (26/607) | -0.084 | -0.01 |
| Poor(%) | 0.38 (2/521) | 0.56 (6/1066) | 0.46 (2/434) | 0.6 (4/607) | -0.032 | -0.024 |
| How often in past week subject exercised |  |  |  |  |  |  |
| Not at all (%) | 15.55 (81/521) | 24.86 (265/1066) | 15.67 (68/434) | 19.58 (138/607) | -0.29 | -0.121 |
| 1 - 2 times (%) | 28.21 (147/521) | 32.08 (342/1066) | 29.26 (127/434) | 28.25 (181/607) | -0.101 | 0.026 |
| 3 - 4 times (%) | 23.99 (125/521) | 22.8 (243/1066) | 25.81 (112/434) | 27.77 (156/607) | 0.034 | -0.055 |
| 5+ times (%) | 32.25 (168/521) | 20.26 (216/1066) | 29.26 (127/434) | 24.4 (132/607) | 0.318 | 0.129 |
| How often in past year has subject had |  |  |  |  |  |  |
| Headaches |  |  |  |  |  |  |
| Never (%) | 10.17 (53/521) | 11.44 (122/1066) | 11.06 (48/434) | 11.05 (70/607) | -0.049 | 0 |
| A few times (%) | 70.44 (367/521) | 67.92 (724/1066) | 70.74 (307/434) | 70.9 (429/607) | 0.066 | -0.004 |
| Once a week (%) | 16.51 (86/521) | 16.14 (172/1066) | 15.9 (69/434) | 14.8 (87/607) | 0.012 | 0.035 |
| Almost every day (%) | 2.5 (13/521) | 3.66 (39/1066) | 1.84 (8/434) | 2.57 (16/607) | -0.084 | -0.053 |
| Every day (%) | 0.38 (2/521) | 0.84 (9/1066) | 0.46 (2/434) | 0.67 (5/607) | -0.077 | -0.035 |
| Unexplained Physical Weakness |  |  |  |  |  |  |
| Never (%) | 61.23 (319/521) | 59.19 (631/1066) | 62.21 (270/434) | 65.75 (398/607) | 0.05 | -0.087 |
| A few times (%) | 34.74 (181/521) | 34.62 (369/1066) | 34.1 (148/434) | 30.12 (184/607) | 0.003 | 0.1 |
| Once a week (%) | 3.84 (20/521) | 4.78 (51/1066) | 3.46 (15/434) | 3.8 (22/607) | -0.057 | -0.021 |
| Almost every day (%) | 0.19 (1/521) | 1.22 (13/1066) | 0.23 (1/434) | 0.33 (3/607) | -0.176 | -0.016 |
| Every day (%) | 0 (0/521) | 0.19 (2/1066) | 0 (0/434) | 0 (0/607) | -0.096 | 0 |
| Dizziness |  |  |  |  |  |  |
| Never (%) | 69.1 (360/521) | 65.38 (697/1066) | 70.05 (304/434) | 70.1 (425/607) | 0.095 | -0.001 |
| A few times (%) | 25.53 (133/521) | 28.61 (305/1066) | 26.73 (116/434) | 25.41 (157/607) | -0.084 | 0.036 |
| Once a week (%) | 4.22 (22/521) | 4.22 (45/1066) | 3 (13/434) | 3.45 (20/607) | 0 | -0.027 |
| Almost every day (%) | 0.77 (4/521) | 1.03 (11/1066) | 0.23 (1/434) | 1.04 (5/607) | -0.034 | -0.105 |
| Every day (%) | 0.38 (2/521) | 0.75 (8/1066) | 0 (0/434) | 0 (0/607) | -0.063 | 0 |
| Muscle or joint pain |  |  |  |  |  |  |
| Never (%) | 11.13 (58/521) | 22.8 (243/1066) | 11.98 (52/434) | 14.78 (111/607) | -0.4 | -0.096 |
| A few times (%) | 50.86 (265/521) | 53.28 (568/1066) | 52.07 (226/434) | 55.05 (337/607) | -0.058 | -0.071 |
| Once a week (%) | 28.6 (149/521) | 19.61 (209/1066) | 28.57 (124/434) | 24.36 (129/607) | 0.246 | 0.115 |
| Almost every day (%) | 7.29 (38/521) | 3.38 (36/1066) | 6.68 (29/434) | 4.38 (23/607) | 0.195 | 0.115 |
| Every day (%) | 2.11 (11/521) | 0.94 (10/1066) | 0.69 (3/434) | 1.44 (7/607) | 0.106 | -0.068 |
| Trouble Sleeping |  |  |  |  |  |  |
| Never (%) | 44.15 (230/521) | 42.21 (450/1066) | 44.93 (195/434) | 45.14 (273/607) | 0.047 | -0.005 |
| A few times (%) | 37.62 (196/521) | 34.05 (363/1066) | 37.79 (164/434) | 36.42 (221/607) | 0.088 | 0.034 |
| Once a week (%) | 11.32 (59/521) | 13.32 (142/1066) | 11.75 (51/434) | 11.66 (71/607) | -0.074 | 0.003 |
| Almost every day (%) | 4.61 (24/521) | 7.22 (77/1066) | 3.92 (17/434) | 5.01 (30/607) | -0.139 | -0.058 |
| Every day (%) | 2.3 (12/521) | 3.19 (34/1066) | 1.61 (7/434) | 1.77 (12/607) | -0.067 | -0.012 |
| Seriously contemplated suicide (%) | 9.4 (49/521) | 11.54 (123/1066) | 7.14 (31/434) | 6.11 (40/607) | -0.085 | 0.041 |
| Desire to go to college |  |  |  |  |  |  |
| 1 - low (%) | 3.26 (17/521) | 6.85 (73/1066) | 2.76 (12/434) | 2.36 (20/607) | -0.212 | 0.024 |
| 2 (%) | 1.34 (7/521) | 4.88 (52/1066) | 1.15 (5/434) | 2.74 (23/607) | -0.279 | -0.125 |
| 3 - medium (%) | 7.49 (39/521) | 13.6 (145/1066) | 6.91 (30/434) | 10.63 (71/607) | -0.253 | -0.154 |
| 4 (%) | 14.01 (73/521) | 17.92 (191/1066) | 14.52 (63/434) | 17.73 (111/607) | -0.13 | -0.107 |
| 5 - high (%) | 73.7 (384/521) | 56.57 (603/1066) | 74.65 (324/434) | 66.47 (381/607) | 0.448 | 0.214 |
| Likelihood to go to college (%) |  |  |  |  |  |  |
| 1 - low (%) | 3.84 (20/521) | 7.69 (82/1066) | 3.46 (15/434) | 3.61 (29/607) | -0.213 | -0.008 |
| 2 (%) | 4.03 (21/521) | 7.5 (80/1066) | 3.92 (17/434) | 3.98 (32/607) | -0.19 | -0.004 |
| 3 - medium (%) | 14.2 (74/521) | 18.95 (202/1066) | 13.36 (58/434) | 16.32 (105/607) | -0.156 | -0.097 |
| 4 (%) | 23.99 (125/521) | 22.51 (240/1066) | 24.19 (105/434) | 23.66 (140/607) | 0.042 | 0.015 |
| 5 - high (%) | 53.74 (280/521) | 43.15 (460/1066) | 55.07 (239/434) | 52.35 (300/607) | 0.254 | 0.065 |
| Ever tried cigarette smoking (%) | 53.55 (279/521) | 57.69 (615/1066) | 49.77 (216/434) | 49.58 (296/607) | -0.099 | 0.005 |
| Smoked regularly (%) | 0.58 (3/521) | 0.56 (6/1066) | 0.46 (2/434) | 0.23 (1/607) | 0.002 | 0.032 |
| Ever drank alcohol (%) | 14.01 (73/521) | 24.02 (256/1066) | 11.29 (49/434) | 12.71 (85/607) | -0.321 | -0.046 |
| How often subject drank alcohol in past year |  |  |  |  |  |  |
| Almost every day (%) | 0.77 (4/521) | 1.41 (15/1066) | 0.46 (2/434) | 0.81 (4/607) | -0.079 | -0.042 |
| 3 - 5 days/week (%) | 2.88 (15/521) | 3.1 (33/1066) | 1.61 (7/434) | 2.4 (13/607) | -0.015 | -0.056 |
| 1 - 2 days/week (%) | 6.33 (33/521) | 7.13 (76/1066) | 4.61 (20/434) | 5.24 (33/607) | -0.038 | -0.031 |
| 2 - 3 days/month (%) | 10.17 (53/521) | 7.41 (79/1066) | 8.53 (37/434) | 5.84 (33/607) | 0.113 | 0.11 |
| Once a month or less (%) | 10.56 (55/521) | 10.6 (113/1066) | 10.14 (44/434) | 8.06 (49/607) | -0.002 | 0.08 |
| Once or twice in past year (%) | 14.4 (75/521) | 15.48 (165/1066) | 14.98 (65/434) | 15.79 (97/607) | -0.036 | -0.027 |
| Never (%) | 9.79 (51/521) | 9.94 (106/1066) | 10.6 (46/434) | 10.45 (59/607) | -0.006 | 0.006 |
| Not applicable (%) | 45.11 (235/521) | 44.93 (479/1066) | 49.08 (213/434) | 51.41 (319/607) | 0.003 | -0.047 |
| How often in past year subject drank 5+ alcoholic drinks in a row |  |  |  |  |  |  |
| Almost every day (%) | 0.77 (4/521) | 0.84 (9/1066) | 0.46 (2/434) | 0.92 (4/607) | -0.01 | -0.062 |
| 3 - 5 days/week (%) | 1.73 (9/521) | 2.35 (25/1066) | 0.69 (3/434) | 0.61 (4/607) | -0.054 | 0.007 |
| 1 - 2 days/week (%) | 4.99 (26/521) | 4.69 (50/1066) | 3.69 (16/434) | 2.73 (19/607) | 0.017 | 0.053 |
| 2 - 3 days/month (%) | 6.91 (36/521) | 6.29 (67/1066) | 6.22 (27/434) | 4.38 (29/607) | 0.03 | 0.088 |
| Once a month or less (%) | 6.14 (32/521) | 5.25 (56/1066) | 5.3 (23/434) | 3.78 (22/607) | 0.045 | 0.077 |
| Once or twice in past year (%) | 7.29 (38/521) | 7.79 (83/1066) | 7.14 (31/434) | 5.09 (37/607) | -0.022 | 0.093 |
| Never (%) | 16.89 (88/521) | 17.92 (191/1066) | 16.36 (71/434) | 20.63 (114/607) | -0.032 | -0.135 |
| Not applicable (%) | 55.28 (288/521) | 54.88 (585/1066) | 60.14 (261/434) | 61.86 (378/607) | 0.008 | -0.035 |
| How much subject agrees that |  |  |  |  |  |  |
| You never get sad |  |  |  |  |  |  |
| Strongly agree (%) | 4.03 (21/521) | 5.63 (60/1066) | 3.69 (16/434) | 6.62 (34/607) | -0.092 | -0.169 |
| Agree (%) | 15.16 (79/521) | 13.13 (140/1066) | 14.75 (64/434) | 15.8 (99/607) | 0.069 | -0.036 |
| Neither agree nor disagree (%) | 19.77 (103/521) | 21.67 (231/1066) | 20.28 (88/434) | 25.55 (147/607) | -0.056 | -0.156 |
| Disagree (%) | 53.17 (277/521) | 48.78 (520/1066) | 53.23 (231/434) | 41.87 (269/607) | 0.105 | 0.271 |
| Strongly disagree (%) | 7.87 (41/521) | 10.79 (115/1066) | 8.06 (35/434) | 10.16 (58/607) | -0.124 | -0.089 |
| You are physically fit |  |  |  |  |  |  |
| Strongly agree (%) | 42.61 (222/521) | 24.95 (266/1066) | 41.94 (182/434) | 42.15 (203/607) | 0.441 | -0.005 |
| Agree (%) | 47.6 (248/521) | 48.69 (519/1066) | 47.93 (208/434) | 44.59 (300/607) | -0.026 | 0.08 |
| Neither agree nor disagree (%) | 8.06 (42/521) | 17.26 (184/1066) | 8.06 (35/434) | 10.65 (80/607) | -0.358 | -0.101 |
| Disagree (%) | 1.73 (9/521) | 8.07 (86/1066) | 2.07 (9/434) | 2.53 (22/607) | -0.413 | -0.03 |
| Strongly disagree (%) | 0 (0/521) | 0.94 (10/1066) | 0 (0/434) | 0.09 (2/607) | -0.216 | -0.02 |
| You feel socially accepted |  |  |  |  |  |  |
| Strongly agree (%) | 38.2 (199/521) | 28.99 (309/1066) | 38.02 (165/434) | 40.41 (212/607) | 0.231 | -0.06 |
| Agree (%) | 54.13 (282/521) | 54.6 (582/1066) | 54.38 (236/434) | 48.46 (330/607) | -0.011 | 0.142 |
| Neither agree nor disagree (%) | 6.14 (32/521) | 11.26 (120/1066) | 6.22 (27/434) | 9.17 (54/607) | -0.231 | -0.133 |
| Disagree (%) | 1.15 (6/521) | 4.78 (51/1066) | 0.92 (4/434) | 1.73 (10/607) | -0.297 | -0.066 |
| Strongly disagree (%) | 0.38 (2/521) | 0.38 (4/1066) | 0.46 (2/434) | 0.23 (1/607) | 0.002 | 0.045 |
| How much subject feels his parents care about him |  |  |  |  |  |  |
| Not at all (%) | 0.19 (1/521) | 0.28 (3/1066) | 0.23 (1/434) | 0.31 (2/607) | -0.023 | -0.02 |
| Very little (%) | 0.77 (4/521) | 0.66 (7/1066) | 0.92 (4/434) | 0.23 (1/607) | 0.016 | 0.097 |
| Somewhat (%) | 2.3 (12/521) | 3.1 (33/1066) | 1.61 (7/434) | 1.94 (13/607) | -0.06 | -0.025 |
| Quite a bit (%) | 10.56 (55/521) | 13.79 (147/1066) | 9.68 (42/434) | 9.33 (69/607) | -0.121 | 0.013 |
| Very much (%) | 85.99 (448/521) | 81.89 (873/1066) | 87.56 (380/434) | 88.08 (521/607) | 0.136 | -0.017 |

**Table G. Comparison of average baseline variables for sport controls and non-sport controls.**

|  | Before Matching | | After Matching | | Standardized Difference | |
| --- | --- | --- | --- | --- | --- | --- |
|  | Sport controls | Non-sport controls | Sport controls | Non-sport controls | Before | After |
|  |  |  |  |  |  |  |
| Age in 2008 (yrs) | 28.94 | 29.13 | 28.94 | 28.96 | -0.108 | -0.016 |
| Weight in 1994-95 (lbs) | 67.88 | 68.13 | 68.02 | 67.76 | -0.015 | 0.016 |
| Height in 1994-95 (cm) | 175.59 | 173 | 175.41 | 173.91 | 0.253 | 0.147 |
| Self-reported race |  |  |  |  |  |  |
| White (%) | 71.64 (437/610) | 73.08 (779/1066) | 71.94 (400/556) | 74.88 (553/741) | -0.037 | -0.075 |
| Black (%) | 21.97 (134/610) | 15.29 (163/1066) | 21.58 (120/556) | 17.22 (124/741) | 0.193 | 0.126 |
| Native American (%) | 1.8 (11/610) | 3.1 (33/1066) | 1.8 (10/556) | 2.23 (18/741) | -0.1 | -0.034 |
| Asian (%) | 5.74 (35/610) | 4.32 (46/1066) | 5.4 (30/556) | 3 (25/741) | 0.073 | 0.123 |
| Other (%) | 3.44 (21/610) | 7.88 (84/1066) | 3.24 (18/556) | 6.41 (49/741) | -0.236 | -0.169 |
| Self-reported rating of general health |  |  |  |  |  |  |
| Excellent (%) | 37.05 (226/610) | 27.39 (292/1066) | 37.05 (206/556) | 35.25 (233/741) | 0.235 | 0.044 |
| Very good (%) | 42.3 (258/610) | 37.99 (405/1066) | 42.81 (238/556) | 38.76 (285/741) | 0.101 | 0.095 |
| Good (%) | 17.21 (105/610) | 28.8 (307/1066) | 16.91 (94/556) | 22.15 (188/741) | -0.328 | -0.149 |
| Fair (%) | 2.95 (18/610) | 5.25 (56/1066) | 2.88 (16/556) | 3.61 (33/741) | -0.139 | -0.044 |
| Poor(%) | 0.33 (2/610) | 0.56 (6/1066) | 0.36 (2/556) | 0.22 (2/741) | -0.042 | 0.024 |
| How often in past week subject exercised |  |  |  |  |  |  |
| Not at all (%) | 20.33 (124/610) | 24.86 (265/1066) | 21.04 (117/556) | 23.23 (181/741) | -0.126 | -0.061 |
| 1 - 2 times (%) | 28.52 (174/610) | 32.08 (342/1066) | 27.52 (153/556) | 31.53 (242/741) | -0.089 | -0.101 |
| 3 - 4 times (%) | 21.48 (131/610) | 22.8 (243/1066) | 21.58 (120/556) | 23.53 (164/741) | -0.037 | -0.054 |
| 5+ times (%) | 29.51 (180/610) | 20.26 (216/1066) | 29.86 (166/556) | 21.7 (154/741) | 0.242 | 0.213 |
| How often in past year has subject had |  |  |  |  |  |  |
| Headaches |  |  |  |  |  |  |
| Never (%) | 11.64 (71/610) | 11.44 (122/1066) | 11.51 (64/556) | 11.81 (83/741) | 0.007 | -0.011 |
| A few times (%) | 65.9 (402/610) | 67.92 (724/1066) | 66.73 (371/556) | 69.71 (519/741) | -0.049 | -0.073 |
| Once a week (%) | 20.16 (123/610) | 16.14 (172/1066) | 19.6 (109/556) | 15.33 (112/741) | 0.119 | 0.126 |
| Almost every day (%) | 1.8 (11/610) | 3.66 (39/1066) | 1.98 (11/556) | 2.67 (23/741) | -0.138 | -0.051 |
| Every day (%) | 0.33 (2/610) | 0.84 (9/1066) | 0.18 (1/556) | 0.48 (4/741) | -0.084 | -0.049 |
| Unexplained Physical Weakness |  |  |  |  |  |  |
| Never (%) | 62.62 (382/610) | 59.19 (631/1066) | 63.13 (351/556) | 65.02 (480/741) | 0.081 | -0.045 |
| A few times (%) | 31.8 (194/610) | 34.62 (369/1066) | 31.47 (175/556) | 31.26 (233/741) | -0.069 | 0.005 |
| Once a week (%) | 4.43 (27/610) | 4.78 (51/1066) | 4.32 (24/556) | 3.37 (24/741) | -0.02 | 0.052 |
| Almost every day (%) | 0.49 (3/610) | 1.22 (13/1066) | 0.54 (3/556) | 0.34 (4/741) | -0.097 | 0.026 |
| Every day (%) | 0.49 (3/610) | 0.19 (2/1066) | 0.54 (3/556) | 0 (0/741) | 0.056 | 0.1 |
| Dizziness |  |  |  |  |  |  |
| Never (%) | 64.92 (396/610) | 65.38 (697/1066) | 67.09 (373/556) | 70.53 (520/741) | -0.011 | -0.083 |
| A few times (%) | 30.98 (189/610) | 28.61 (305/1066) | 29.68 (165/556) | 24.42 (185/741) | 0.059 | 0.132 |
| Once a week (%) | 2.62 (16/610) | 4.22 (45/1066) | 2.16 (12/556) | 4.02 (28/741) | -0.105 | -0.122 |
| Almost every day (%) | 0.98 (6/610) | 1.03 (11/1066) | 0.72 (4/556) | 0.97 (7/741) | -0.006 | -0.029 |
| Every day (%) | 0.33 (2/610) | 0.75 (8/1066) | 0.36 (2/556) | 0.06 (1/741) | -0.071 | 0.05 |
| Muscle or joint pain |  |  |  |  |  |  |
| Never (%) | 15.57 (95/610) | 22.8 (243/1066) | 16.37 (91/556) | 20.04 (161/741) | -0.216 | -0.11 |
| A few times (%) | 53.44 (326/610) | 53.28 (568/1066) | 53.78 (299/556) | 54.9 (411/741) | 0.004 | -0.026 |
| Once a week (%) | 21.8 (133/610) | 19.61 (209/1066) | 21.76 (121/556) | 20.38 (142/741) | 0.062 | 0.039 |
| Almost every day (%) | 7.87 (48/610) | 3.38 (36/1066) | 6.83 (38/556) | 3.6 (21/741) | 0.212 | 0.153 |
| Every day (%) | 1.15 (7/610) | 0.94 (10/1066) | 1.26 (7/556) | 1.08 (6/741) | 0.023 | 0.02 |
| Trouble Sleeping |  |  |  |  |  |  |
| Never (%) | 44.26 (270/610) | 42.21 (450/1066) | 45.5 (253/556) | 46.42 (332/741) | 0.047 | -0.021 |
| A few times (%) | 35.74 (218/610) | 34.05 (363/1066) | 35.61 (198/556) | 35.81 (265/741) | 0.041 | -0.005 |
| Once a week (%) | 11.97 (73/610) | 13.32 (142/1066) | 11.51 (64/556) | 11.38 (90/741) | -0.047 | 0.005 |
| Almost every day (%) | 6.72 (41/610) | 7.22 (77/1066) | 6.29 (35/556) | 4.66 (41/741) | -0.023 | 0.074 |
| Every day (%) | 1.15 (7/610) | 3.19 (34/1066) | 1.08 (6/556) | 1.74 (13/741) | -0.174 | -0.056 |
| Seriously contemplated suicide (%) | 7.87 (48/610) | 11.54 (123/1066) | 7.37 (41/556) | 7.93 (62/741) | -0.146 | -0.022 |
| Desire to go to college |  |  |  |  |  |  |
| 1 - low (%) | 2.95 (18/610) | 6.85 (73/1066) | 2.7 (15/556) | 3.88 (34/741) | -0.222 | -0.067 |
| 2 (%) | 2.95 (18/610) | 4.88 (52/1066) | 3.06 (17/556) | 3.12 (31/741) | -0.119 | -0.004 |
| 3 - medium (%) | 9.67 (59/610) | 13.6 (145/1066) | 10.25 (57/556) | 10.69 (89/741) | -0.144 | -0.016 |
| 4 (%) | 11.64 (71/610) | 17.92 (191/1066) | 11.15 (62/556) | 17.25 (139/741) | -0.21 | -0.204 |
| 5 - high (%) | 72.46 (442/610) | 56.57 (603/1066) | 72.66 (404/556) | 64.94 (446/741) | 0.393 | 0.191 |
| Likelihood to go to college (%) |  |  |  |  |  |  |
| 1 - low (%) | 4.1 (25/610) | 7.69 (82/1066) | 3.96 (22/556) | 4.9 (43/741) | -0.184 | -0.048 |
| 2 (%) | 4.43 (27/610) | 7.5 (80/1066) | 4.68 (26/556) | 4 (41/741) | -0.156 | 0.034 |
| 3 - medium (%) | 11.31 (69/610) | 18.95 (202/1066) | 10.43 (58/556) | 16.41 (129/741) | -0.255 | -0.199 |
| 4 (%) | 25.41 (155/610) | 22.51 (240/1066) | 25.36 (141/556) | 23.16 (179/741) | 0.077 | 0.059 |
| 5 - high (%) | 54.26 (331/610) | 43.15 (460/1066) | 55.22 (307/556) | 51.41 (347/741) | 0.256 | 0.088 |
| Ever tried cigarette smoking (%) | 53.28 (325/610) | 57.69 (615/1066) | 51.98 (289/556) | 50.88 (378/741) | -0.102 | 0.025 |
| Smoked regularly (%) | 0.49 (3/610) | 0.56 (6/1066) | 0.36 (2/556) | 0.24 (2/741) | -0.011 | 0.018 |
| Ever drank alcohol (%) | 14.26 (87/610) | 24.02 (256/1066) | 12.77 (71/556) | 15.21 (121/741) | -0.296 | -0.074 |
| How often subject drank alcohol in past year |  |  |  |  |  |  |
| Almost every day (%) | 0.82 (5/610) | 1.41 (15/1066) | 0.54 (3/556) | 0.72 (5/741) | -0.067 | -0.021 |
| 3 - 5 days/week (%) | 3.61 (22/610) | 3.1 (33/1066) | 3.42 (19/556) | 2.71 (19/741) | 0.032 | 0.044 |
| 1 - 2 days/week (%) | 6.39 (39/610) | 7.13 (76/1066) | 5.94 (33/556) | 4.81 (42/741) | -0.034 | 0.052 |
| 2 - 3 days/month (%) | 6.56 (40/610) | 7.41 (79/1066) | 6.29 (35/556) | 5.8 (42/741) | -0.039 | 0.022 |
| Once a month or less (%) | 11.97 (73/610) | 10.6 (113/1066) | 11.87 (66/556) | 10.28 (77/741) | 0.049 | 0.057 |
| Once or twice in past year (%) | 14.1 (86/610) | 15.48 (165/1066) | 13.13 (73/556) | 15.81 (115/741) | -0.045 | -0.087 |
| Never (%) | 10.49 (64/610) | 9.94 (106/1066) | 10.25 (57/556) | 9.61 (74/741) | 0.021 | 0.024 |
| Not applicable(%) | 46.07 (281/610) | 44.93 (479/1066) | 48.56 (270/556) | 50.25 (367/741) | 0.023 | -0.034 |
| How often in past year subject drank 5+ alcoholic drinks in a row |  |  |  |  |  |  |
| Almost every day (%) | 0.49 (3/610) | 0.84 (9/1066) | 0.36 (2/556) | 0.63 (4/741) | -0.052 | -0.04 |
| 3 - 5 days/week (%) | 2.46 (15/610) | 2.35 (25/1066) | 2.52 (14/556) | 0.91 (9/741) | 0.008 | 0.12 |
| 1 - 2 days/week (%) | 5.41 (33/610) | 4.69 (50/1066) | 5.4 (30/556) | 3.9 (30/741) | 0.037 | 0.078 |
| 2 - 3 days/month (%) | 3.61 (22/610) | 6.29 (67/1066) | 3.06 (17/556) | 4.5 (37/741) | -0.148 | -0.08 |
| Once a month or less (%) | 6.23 (38/610) | 5.25 (56/1066) | 5.94 (33/556) | 4.72 (32/741) | 0.048 | 0.059 |
| Once or twice in past year (%) | 9.51 (58/610) | 7.79 (83/1066) | 8.81 (49/556) | 5.7 (47/741) | 0.069 | 0.126 |
| Never (%) | 15.57 (95/610) | 17.92 (191/1066) | 14.93 (83/556) | 19.78 (141/741) | -0.073 | -0.151 |
| Not applicable (%) | 56.72 (346/610) | 54.88 (585/1066) | 58.99 (328/556) | 59.86 (441/741) | 0.037 | -0.018 |
| How much subject agrees that |  |  |  |  |  |  |
| You never get sad |  |  |  |  |  |  |
| Strongly agree (%) | 4.92 (30/610) | 5.63 (60/1066) | 5.04 (28/556) | 6.29 (42/741) | -0.037 | -0.065 |
| Agree (%) | 15.25 (93/610) | 13.13 (140/1066) | 16.37 (91/556) | 15.9 (115/741) | 0.069 | 0.015 |
| Neither agree nor disagree (%) | 19.51 (119/610) | 21.67 (231/1066) | 19.42 (108/556) | 21.97 (161/741) | -0.062 | -0.073 |
| Disagree (%) | 51.31 (313/610) | 48.78 (520/1066) | 50.54 (281/556) | 45.97 (351/741) | 0.058 | 0.105 |
| Strongly disagree (%) | 8.85 (54/610) | 10.79 (115/1066) | 8.63 (48/556) | 9.86 (72/741) | -0.076 | -0.048 |
| You are physically fit [H1PF31] |  |  |  |  |  |  |
| Strongly agree (%) | 42.62 (260/610) | 24.95 (266/1066) | 41.37 (230/556) | 37.17 (222/741) | 0.428 | 0.102 |
| Agree (%) | 45.41 (277/610) | 48.69 (519/1066) | 46.22 (257/556) | 48.89 (363/741) | -0.075 | -0.061 |
| Neither agree nor disagree (%) | 9.02 (55/610) | 17.26 (184/1066) | 9.35 (52/556) | 11.11 (112/741) | -0.295 | -0.063 |
| Disagree (%) | 2.46 (15/610) | 8.07 (86/1066) | 2.7 (15/556) | 2.58 (40/741) | -0.317 | 0.007 |
| Strongly disagree (%) | 0.33 (2/610) | 0.94 (10/1066) | 0.36 (2/556) | 0.25 (4/741) | -0.096 | 0.016 |
| You feel socially accepted |  |  |  |  |  |  |
| Strongly agree (%) | 36.72 (224/610) | 28.99 (309/1066) | 37.23 (207/556) | 34.92 (228/741) | 0.188 | 0.056 |
| Agree (%) | 54.26 (331/610) | 54.6 (582/1066) | 54.32 (302/556) | 54.3 (419/741) | -0.008 | 0 |
| Neither agree nor disagree (%) | 6.72 (41/610) | 11.26 (120/1066) | 6.29 (35/556) | 8.11 (68/741) | -0.189 | -0.076 |
| Disagree (%) | 1.97 (12/610) | 4.78 (51/1066) | 2.16 (12/556) | 2.49 (25/741) | -0.192 | -0.022 |
| Strongly disagree (%) | 0 (0/610) | 0.38 (4/1066) | 0 (0/556) | 0.18 (1/741) | -0.12 | -0.058 |
| How much subject feels his parents care about him |  |  |  |  |  |  |
| Not at all (%) | 0.49 (3/610) | 0.28 (3/1066) | 0.36 (2/556) | 0.27 (2/741) | 0.037 | 0.016 |
| Very little (%) | 0.49 (3/610) | 0.66 (7/1066) | 0.54 (3/556) | 0.37 (4/741) | -0.026 | 0.026 |
| Somewhat (%) | 1.8 (11/610) | 3.1 (33/1066) | 1.62 (9/556) | 1.69 (13/741) | -0.1 | -0.006 |
| Quite a bit (%) | 11.48 (70/610) | 13.79 (147/1066) | 11.15 (62/556) | 11.48 (99/741) | -0.081 | -0.012 |
| Very much (%) | 85.41 (521/610) | 81.89 (873/1066) | 86.15 (479/556) | 85.85 (620/741) | 0.111 | 0.009 |

**Table H. Effects of playing football on secondary outcomes compared to sport controls.**

| Outcome | Effect (95% CI) | Small/Large Effect Cut-off | Unadjusted P Value (Adjusted P Value) |
| --- | --- | --- | --- |
| General Health Outcomes |  |  |  |
| Daily Smoker (y/n) | 0.8 (0.55, 1.17) | 1.50 / 5.00 | 0.26 (0.7) |
| Physically Active in 2008 (y/n) | 0.97 (0.59, 1.57) | 1.50 / 5.00 | 0.89 (0.93) |
| Diagnosis of following after age 18 |  |  |  |
| High cholesterol or triglycerides (y/n) | 1.68 (0.96, 2.95) | 1.50 / 5.00 | 0.07 (0.7) |
| High Blood Pressure or Hypertension (y/n) | 1.35 (0.81, 2.25) | 1.50 / 5.00 | 0.25 (0.7) |
| High blood sugar or diabetes (y/n) | 0.76 (0.18, 3.3) | 1.50 / 5.00 | 0.71 (0.91) |
| Heart disease (y/n) | 0.77 (0.06, 9.49) | 1.50 / 5.00 | 0.84 (0.91) |
| Migraine Headaches (y/n) | 1.04 (0.42, 2.57) | 1.50 / 5.00 | 0.94 (0.94) |
| Depression (y/n) | 0.55 (0.27, 1.15) | 1.50 / 5.00 | 0.11 (0.7) |
| PTSD (y/n) | 1.33 (0.3, 5.96) | 1.50 / 5.00 | 0.71 (0.91) |
| Anxiety or Panic Disorder (y/n) | 0.86 (0.42, 1.73) | 1.50 / 5.00 | 0.67 (0.91) |
| Seriously contemplated suicide in past year (y/n) | 0.7 (0.29, 1.7) | 1.50 / 5.00 | 0.43 (0.91) |
| Substance Dependence/Abuse Outcomes |  |  |  |
| Nicotine (y/n) | 0.64 (0.38, 1.08) | 1.50 / 5.00 | 0.1 (0.7) |
| Alcohol (y/n) | 1.11 (0.8, 1.53) | 1.50 / 5.00 | 0.54 (0.91) |
| Cannabis (y/n) | 0.75 (0.46, 1.21) | 1.50 / 5.00 | 0.23 (0.7) |
| Personality Scale Scores |  |  |  |
| Cohen Perceived Stress | -0.2 (-0.7, 0.24) | 0.87 / 3.49 | 0.27 (0.7) |
| Anxiety Scale | 0.11 (-0.3, 0.51) | 0.55 / 2.21 | 0.51 (0.91) |
| Anger/Hostility Scale | 0.11 (-0.31, 0.51) | 0.54 / 2.17 | 0.66 (0.91) |
| Optimism Scale | 0.2 (-0.11, 0.44) | 0.44 / 1.76 | 0.26 (0.7) |
| “Big 5” Personality Traits |  |  |  |
| Agreeableness | -0.13 (-0.52, 0.23) | 0.5 / 2.01 | 0.49 (0.91) |
| Conscientiousness | 0.07 (-0.34, 0.43) | 0.49 / 1.94 | 0.75 (0.91) |
| Extraversion | 0.04 (-0.4, 0.5) | 0.61 / 2.44 | 0.79 (0.91) |
| Neuroticism | -0.24 (-0.58, 0.1) | 0.5 / 1.98 | 0.19 (0.7) |
| Openness | -0.17 (-0.55, 0.32) | 1 / 3.98 | 0.76 (0.91) |

**Table I. Effects of playing football on secondary outcomes compared to non-sport controls**

| Outcome | Effect (95% CI) | Small/Large Effect Cut-off | Unadjusted P Value (Adjusted P Value) |
| --- | --- | --- | --- |
| General Health Outcomes |  |  |  |
| Daily Smoker (y/n) | 0.63 (0.45, 0.88) | 1.50 / 5.00 | 0.006 (0.07) |
| Physically Active in 2008 (y/n) | 0.83 (0.57, 1.19) | 1.50 / 5.00 | 0.31 (0.55) |
| Diagnosis of following after age 18 |  |  |  |
| High cholesterol or triglycerides (y/n) | 1.1 (0.68, 1.77) | 1.50 / 5.00 | 0.7 (0.95) |
| High Blood Pressure or Hypertension (y/n) | 0.85 (0.56, 1.28) | 1.50 / 5.00 | 0.43 (0.66) |
| High blood sugar or diabetes (y/n) | 0.27 (0.09, 0.81) | 1.50 / 5.00 | 0.02 (0.11) |
| Migraine Headaches (y/n) | 0.52 (0.25, 1.1) | 1.50 / 5.00 | 0.09 (0.29) |
| Depression (y/n) | 0.55 (0.31, 0.97) | 1.50 / 5.00 | 0.04 (0.18) |
| PTSD (y/n) | 0.61 (0.23, 1.61) | 1.50 / 5.00 | 0.31 (0.55) |
| Anxiety or Panic Disorder (y/n) | 0.93 (0.53, 1.63) | 1.50 / 5.00 | 0.8 (0.96) |
| Seriously contemplated suicide in past year (y/n) | 0.62 (0.32, 1.2) | 1.50 / 5.00 | 0.16 (0.36) |
| Substance Dependence/Abuse Outcomes |  |  |  |
| Nicotine (y/n) | 0.67 (0.44, 1.01) | 1.50 / 5.00 | 0.06 (0.21) |
| Alcohol (y/n) | 1.23 (0.93, 1.63) | 1.50 / 5.00 | 0.15 (0.36) |
| Cannabis (y/n) | 1.06 (0.75, 1.5) | 1.50 / 5.00 | 0.74 (0.95) |
| Personality Scale Scores |  |  |  |
| Cohen Perceived Stress | 0.05 (-0.31, 0.37) | 0.8 / 3.21 | 0.88 (0.96) |
| Anxiety Scale | -0.04 (-0.41, 0.37) | 0.95 / 3.82 | 0.96 (1) |
| Anger/Hostility Scale | -0.08 (-0.48, 0.25) | 0.57 / 2.28 | 0.59 (0.85) |
| Optimism Scale | 0.23 (-0.06, 0.44) | 0.71 / 2.82 | 0.12 (0.35) |
| “Big 5” Personality Traits |  |  |  |
| Agreeableness | -0.15 (-0.53, 0.13) | 0.87 / 3.5 | 0.33 (0.55) |
| Conscientiousness | 0.4 (0.08, 0.76) | 0.89 / 3.57 | 0.01 (0.11) |
| Extraversion | 0.16 (-0.24, 0.57) | 0.8 / 3.21 | 0.31 (0.55) |
| Neuroticism | -0.07 (-0.37, 0.3) | 0.74 / 2.97 | 0.85 (0.96) |
| Openness | -0.72 (-1.16, -0.32) | 1.61 / 6.45 | 0.002 (0.04) |

**Table J. Effects of playing a non-collision sport vs not playing any sport on secondary outcomes.**

| Outcome | Effect (95% CI) | Small/Large Effect Cut-off | Unadjusted P Value (Adjusted P Value) |
| --- | --- | --- | --- |
| General Health Outcomes |  |  |  |
| Daily Smoker (y/n) | 0.96 (0.73, 1.27) | 1.50 / 5.00 | 0.8 (0.9) |
| Physically Active in 2008 (y/n) | 0.74 (0.52, 1.05) | 1.50 / 5.00 | 0.09 (0.42) |
| Diagnosis of following after age 18 |  |  |  |
| High cholesterol or triglycerides (y/n) | 0.71 (0.46, 1.11) | 1.50 / 5.00 | 0.13 (0.42) |
| High Blood Pressure or Hypertension (y/n) | 0.97 (0.68, 1.38) | 1.50 / 5.00 | 0.86 (0.9) |
| High blood sugar or diabetes (y/n) | 0.58 (0.26, 1.31) | 1.50 / 5.00 | 0.19 (0.42) |
| Heart disease (y/n) | 0.6 (0.11, 3.37) | 1.50 / 5.00 | 0.56 (0.78) |
| Migraine Headaches (y/n) | 0.62 (0.32, 1.18) | 1.50 / 5.00 | 0.14 (0.42) |
| Depression (y/n) | 0.72 (0.46, 1.13) | 1.50 / 5.00 | 0.15 (0.42) |
| PTSD (y/n) | 0.55 (0.21, 1.43) | 1.50 / 5.00 | 0.22 (0.42) |
| Anxiety or Panic Disorder (y/n) | 1.05 (0.65, 1.71) | 1.50 / 5.00 | 0.84 (0.9) |
| Seriously contemplated suicide in past year (y/n) | 0.82 (0.48, 1.43) | 1.50 / 5.00 | 0.49 (0.75) |
| Substance Dependence/Abuse Outcomes |  |  |  |
| Nicotine (y/n) | 0.78 (0.55, 1.12) | 1.50 / 5.00 | 0.18 (0.42) |
| Alcohol (y/n) | 1.15 (0.9, 1.48) | 1.50 / 5.00 | 0.25 (0.45) |
| Cannabis (y/n) | 1.1 (0.79, 1.52) | 1.50 / 5.00 | 0.57 (0.78) |
| Personality Scale Scores |  |  |  |
| Cohen Perceived Stress | 0.24 (-0.09, 0.53) | 0.92 / 3.67 | 0.18 (0.42) |
| Anxiety Scale | -0.17 (-0.55, 0.22) | 1.01 / 4.05 | 0.31 (0.52) |
| Anger/Hostility Scale | -0.19 (-0.51, 0.15) | 0.75 / 3.02 | 0.22 (0.42) |
| Optimism Scale | -0.04 (-0.32, 0.26) | 0.81 / 3.24 | 0.92 (0.92) |
| “Big 5” Personality Traits |  |  |  |
| Agreeableness | -0.15 (-0.4, 0.23) | 0.94 / 3.75 | 0.73 (0.9) |
| Conscientiousness | 0.36 (-0.02, 0.68) | 0.95 / 3.81 | 0.03 (0.39) |
| Extraversion | 0.3 (-0.09, 0.64) | 0.91 / 3.63 | 0.12 (0.42) |
| Neuroticism | 0.02 (-0.3, 0.35) | 0.85 / 3.42 | 0.8 (0.9) |
| Openness | -0.94 (-1.61, -0.4) | 1.73 / 6.91 | P<.001 (0.02) |
